# A Subtype of Ultrasonic Vocalizations during Palatable Food Consumption in Rats Identified by Machine Learning-Assisted Classification

**DOI:** 10.1523/ENEURO.0356-25.2026

**Published:** 2026-08-06

**Authors:** Koshi Murata, Yuki Ikedo, Takashi Ryoke, Kazuki Shiotani, Hiroyuki Manabe, Kazuki Kuroda, Hitoshi Yoshimura, Yugo Fukazawa

**Affiliations:** ^1^Division of Brain Structure and Function, Faculty of Medical Sciences, University of Fukui, Fukui 910-1193, Japan; ^2^Life Science Innovation Center, University of Fukui, Fukui 910-1193, Japan; ^3^Department of Dentistry and Oral Surgery, Faculty of Medical Sciences, University of Fukui, Fukui 910-1193, Japan; ^4^Department of Applied Life Sciences, Graduate School of Bioagricultural Sciences, Nagoya University, Nagoya 464-8601, Japan; ^5^Department of Neurophysiology, Nara Medical University, Nara 634-8521, Japan

**Keywords:** emotion, feeding behavior, machine learning classification, opioid system, positive affect, ultrasonic vocalization

## Abstract

Identifying behavioral and physiological responses to rewarding stimuli is essential for understanding positive emotional states in animals and for investigating their neural basis. Ultrasonic vocalizations (USVs) in rats are modulated by various behavioral contexts and are thought to represent affective-like states. However, their diversity during palatable food consumption remains underexplored. In this study, we investigated acoustic features of USVs in male rats consuming chocolate, a highly palatable food. Using a machine learning–assisted logistic regression model trained on spectrogram features, we identified a distinct USV subtype—the 40 kHz inverted-U type—that was selectively emitted during chocolate consumption. The emission of this subtype was tightly time-locked to chocolate-feeding behavior. Systemic administration of naloxone, an opioid receptor antagonist, significantly reduced the emission of the 40 kHz inverted-U USVs during chocolate intake. Direct comparison with standard food pellet consumption revealed that 40 kHz flat USVs occurred at comparable levels across both conditions, whereas 40 kHz inverted-U USVs were significantly more frequent during chocolate consumption. These findings suggest that these USVs may reflect internal states associated with palatable feeding and are under modulation by the endogenous opioid system. Moreover, our data demonstrate the utility of machine learning for high-throughput, objective classification of USV subtypes. This framework provides a promising approach for decoding emotion-related vocal expressions in rats and highlights the potential of specific USVs as behavioral readouts for studying the neurobiology of positive emotions.

## Significance Statement

Ultrasonic vocalizations (USVs) are high-frequency calls produced by rats known to correlate with emotional and motivational states, yet their diversity and functional relevance remain underexplored. In this study, we identified a novel 40 kHz inverted-U USV subtype associated with chocolate consumption. These calls were closely tied to feeding behavior and were selectively suppressed by an opioid receptor antagonist, suggesting modulation by the brain's reward system. Using machine learning–assisted classification, we established a robust method to detect these vocalizations. Our findings highlight the potential of specific USVs as noninvasive, real-time behavioral indicators of internal states, offering new tools for studying affect, reward, and communication in animal models.

## Introduction

To understand the neurobiological mechanisms that underlie positive emotions, it is essential to identify behavioral and physiological responses to rewarding external stimuli, which in turn enables objective and quantitative assessment of such emotional states. Food consumption is accompanied by positive emotions in both animals and humans, particularly when the food is highly palatable, such as sweet-tasting foods indicating high nutritional value. These positive emotional experiences are closely associated with learning and motivation, particularly when stimuli reinforce future behaviors (Nguyen et al., 2021).

In animal studies, food palatability and associated positive emotions are frequently evaluated using preference tests, such as food choice paradigms comparing intake between two or more options (Rainwater and Güler, 2022; Cawthon and Spector, 2023). However, preference tests are indirect measures: they assess palatability and emotional valence only through differences in cumulative food intake over time. To better understand the affective component of palatable food consumption, it is important to capture the immediate behavioral and physiological responses that occur at the moment of feeding. A commonly used method for real-time assessment of positive emotions is the taste reactivity test, in which animals receive passive intraoral infusions of sweet solutions (e.g., sucrose) via implanted oral cannulas and their facial and bodily responses are analyzed (Berridge, 2000). In this test, specific ingestive responses are defined as hedonic “liking.” However, this method is invasive and requires physical restraint for cannula connection. Moreover, it is limited to liquid taste stimuli and cannot assess emotional responses to solid foods. Therefore, more versatile, noninvasive, real-time measures of positive emotional states during food consumption in animals are needed to further investigate the underlying neural mechanisms.

Ultrasonic vocalizations (USVs) are thought to reflect positive and negative affective-like states in rats, as they occur in various behavioral contexts including social interaction, mating, play, and exposure to rewarding stimuli (Knutson et al., 2002; Portfors, 2007; Burgdorf et al., 2008; Brudzynski, 2015; Schwarting, 2023). These vocalizations vary in frequency, duration, and contour shape and may reflect the animal's internal state or behavioral intent. Among these, 50 kHz USVs (frequency range, 30–90 kHz; duration, 20–100 ms) have been frequently observed during approach behaviors and active engagement with stimuli, including food anticipation and consumption (Takahashi et al., 2010; Brenes and Schwarting, 2014; Buck et al., 2014; Champeil-Potokar et al., 2023), suggesting an association with motivational and appetitive states (Wright et al., 2010; Buck et al., 2014; Wöhr, 2017). Consumption of a standard laboratory diet elicits a particular subtype of USVs, 40 kHz flat-shaped USVs (Takahashi et al., 2010; Champeil-Potokar et al., 2023). Additionally, intake of a more palatable substance (sweetened condensed milk) induces a greater number of 50 kHz USVs during both consumption and cue-induced anticipation compared with tap water intake (Brenes and Schwarting, 2014), raising the possibility that food palatability modulates vocal output. However, it remains unclear whether specific USV subtypes with distinct acoustic features are selectively emitted during the consumption of highly palatable foods.

To address this question, we examined whether rats emit distinguishable subtypes of USVs when consuming a highly palatable food, as compared with baseline conditions without access to that food. We used sweet milk chocolate as a highly palatable stimulus, as rats exhibit a strong preference for it over standard laboratory chow, even in the absence of food deprivation (Castro and Berridge, 2014). We compared the spectrograms of USVs emitted during chocolate-access periods with those emitted during baseline periods. Using machine learning–assisted classification of spectrographic features, we constructed a logistic regression model to perform binary classification of USVs associated with chocolate presentation versus nonchocolate conditions. Through a combination of machine learning classification and conventional visual assessment, we identified a subtype of 40 kHz inverted-U USVs that were selectively emitted during chocolate consumption. Furthermore, systemic administration of the opioid receptor antagonist naloxone reduced the emission of these USVs, suggesting involvement of the endogenous opioid system. These findings suggest that food palatability may influence vocal expression in rats and raise the possibility that specific USV patterns could serve as real-time behavioral markers of emotional states during palatable feeding.

## Materials and Methods

### Ethical statements

All animal experiments were approved by the Experimental Animal Research Committee of the University of Fukui (approval numbers, R03015, R04014, R05011, and R06007). This study was performed in accordance with relevant guidelines and regulations.

### Animals

Eight-week-old male Sprague Dawley rats were obtained from Japan SLC and housed in our animal facility for at least 1 week before the beginning of the USV recording. A total of 80 rats were used in the experiments: 20 rats (10 pairs) for the training dataset, 22 rats (11 pairs) for the test dataset (empty group), 22 rats (11 pairs) for the test dataset (chocolate group) followed by pharmacological experiments, and 16 rats (8 pairs) for the regular chow-feeding dataset. The rats were housed in pairs in plastic cages (33 × 38 × 18 cm, CL−0106-2, CLEA Japan) with paper bedding (Japan SLC) and wire cage tops. These pairings were maintained throughout the experiment, with each pair housed together in the same home cage, including during all recording sessions. They had *ad libitum* access to pellet food (DC-8, Oriental Yeast), except during USV recording sessions, with water available at all times. The rats were kept on a reverse 12–12 h light/dark cycle, with experiments conducted during the dark phase, 6–9 h (ZT18–21) after lights-off for the training and test datasets, and 0–2 h after lights-off (ZT12–14) for the regular chow-feeding dataset.

### Experimental design

**Figure 1.**
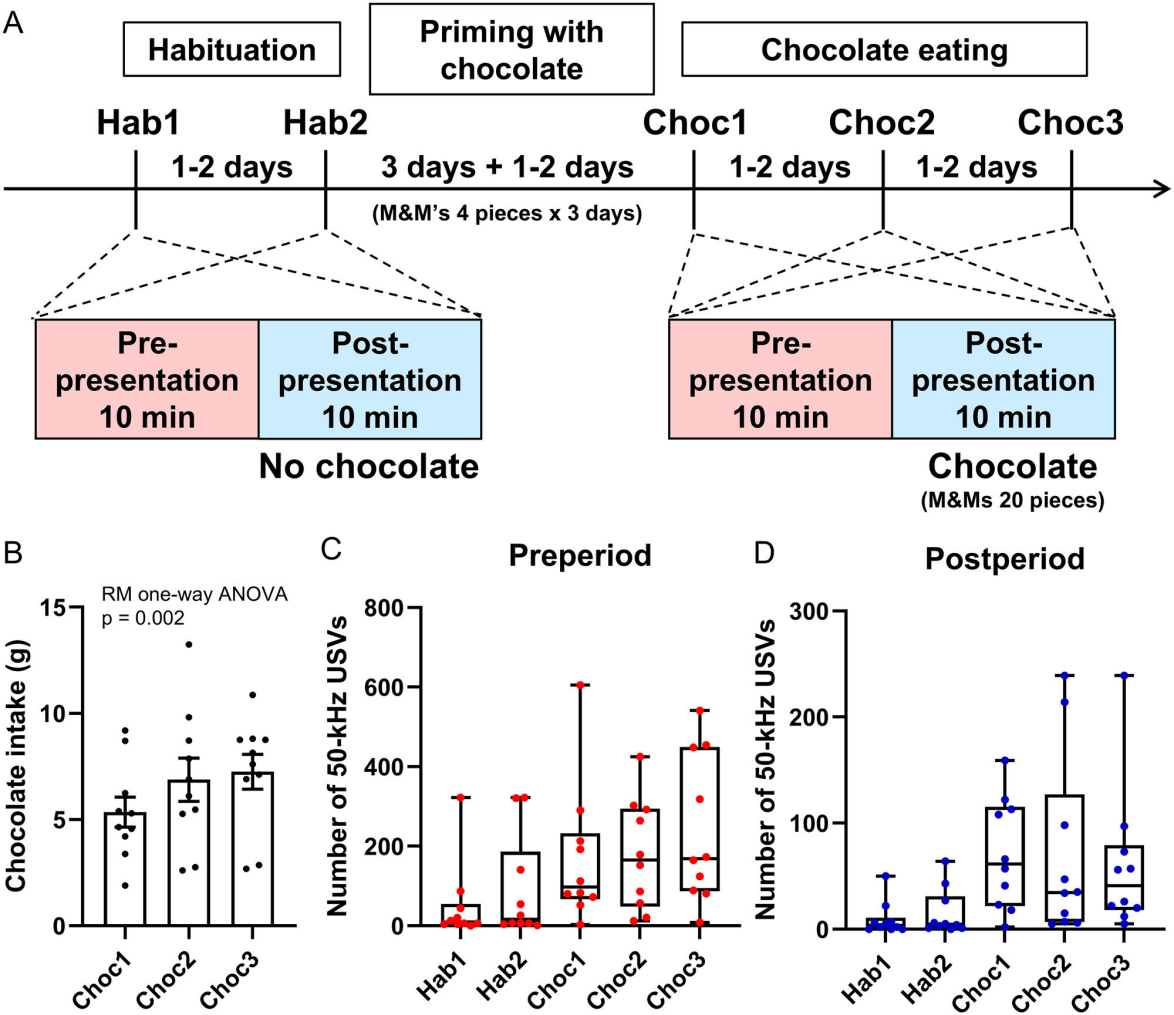
Emission of 50 kHz USVs by pair-housed adult male rats during chocolate consumption. ***A***, Schedule of USV recordings to obtain the training dataset for machine learning–based USV classification. Habituation sessions (hab1 and hab2) were conducted to acclimate the rats to the recording environment and to record USVs in the environment before priming with chocolate. Chocolate presentation sessions (choc1, choc2, and choc3) were conducted to record USVs before and during chocolate consumption. Each session consisted of two 10 min periods: prechocolate presentation period (preperiod) and postchocolate presentation period (postperiod). Intervals between recording sessions lasted 1–2 d. The priming phase for chocolate consumption was set between the habituation and chocolate presentation sessions, during which rats were provided with four pieces (∼4 g) of M&M's milk chocolate once per day for 3 consecutive days in their home environment. The interval between the last day of priming and the first chocolate presentation session (choc1) was also 1–2 d. ***B***, Amount of chocolate intake during the postperiod. ***C, D***, Number of 50 kHz USVs during the pre- (***C***) and postchocolate (***D***) periods (*n* = 10 rat pairs). Hab1, Hab2, habituation sessions 1 and 2; Choc1, Choc2, Choc3, chocolate presentation sessions 1, 2, and 3.

**Figure 2.**
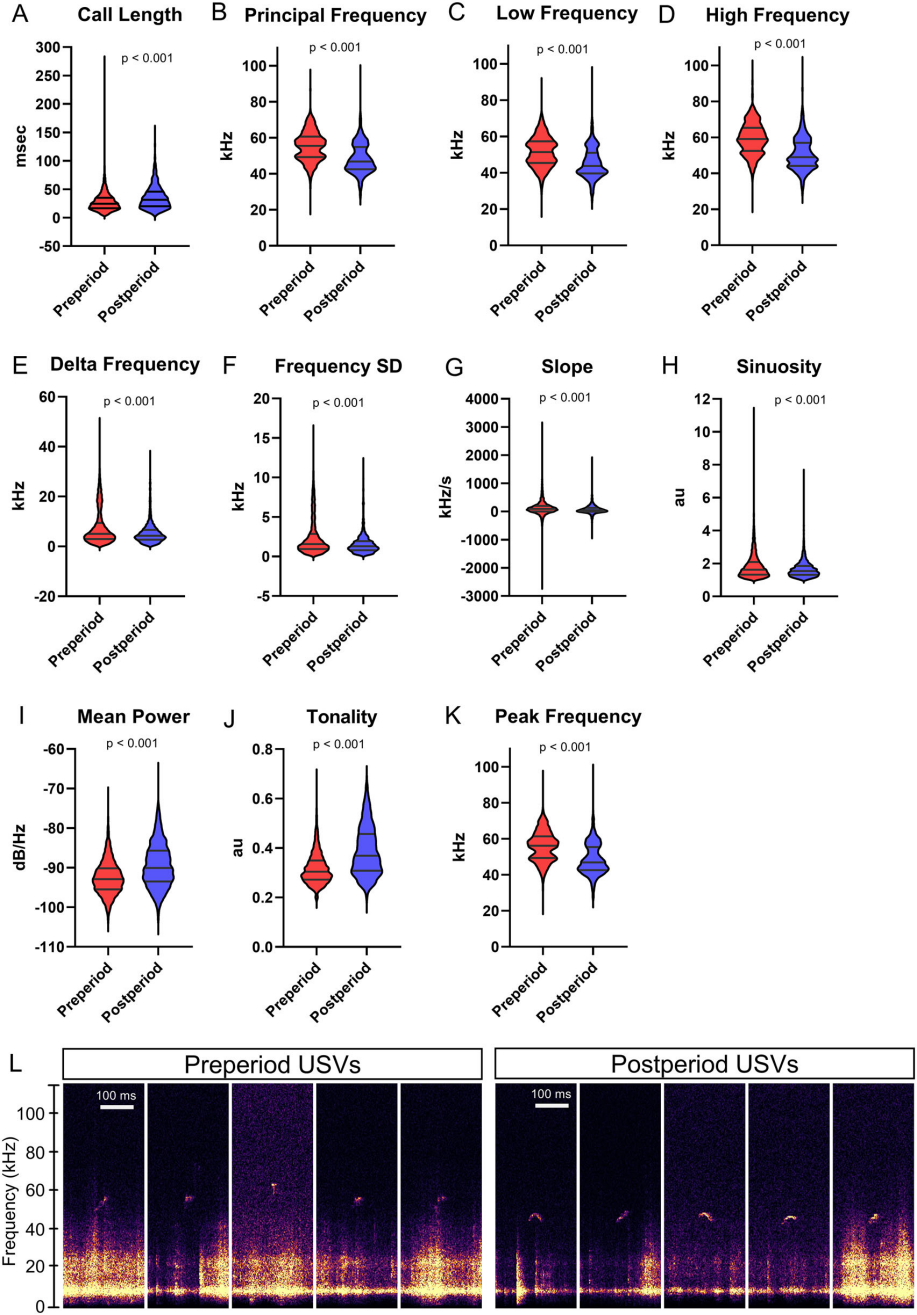
Comparison of USV spectrograms between pre- and postperiods of chocolate delivery. ***A–K***, Violin plots representing the differences in 11 spectrogram parameters between USVs in the pre- and postperiods. USVs from a total of 10 rat pairs and three sessions (choc1, choc2, and choc3) were pooled. The total number of calls was 5,893 for the preperiod and 2,016 for the postperiod. ***A***, Call length, (***B***) principal frequency, (***C***) low frequency, (***D***) high frequency, (***E***) delta frequency, (***F***) standard deviation of frequency, (***G***) slope, (***H***) sinuosity, (***I***) mean power, (***J***) tonality, (***K***) peak frequency. *p* values were obtained from Mann–Whitney *U* tests. In panels ***A–K***, individual calls were initially treated as the statistical unit. To address potential nonindependence of calls within rat pairs, additional analyses treating the rat pair as the experimental unit are reported in Extended Data Figure 2-1. ***L***, Representative spectrograms of the USVs from the pre- and postperiods. The five USVs with the closest Euclidean distances to median values of the 11 spectrogram parameters were selected from all USVs.

**Figure 3.**
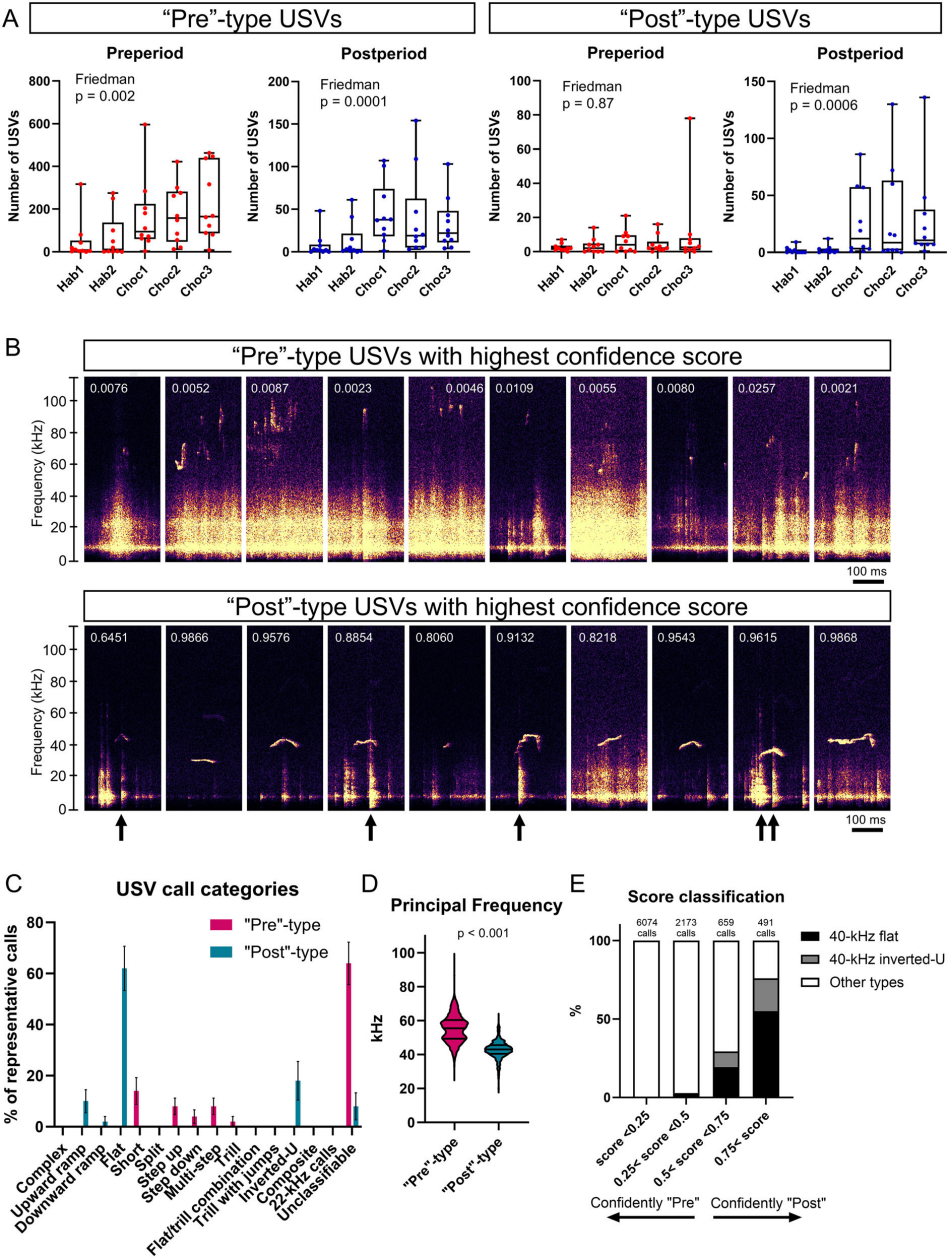
Logistic regression model-assisted identification of 40 kHz flat and 40 kHz inverted-U types as USVs emitted during chocolate consumption. ***A***, Number of “pre”- and “post”-type USVs in the pre- and postperiods of each session (*n* = 10 rat pairs). ***B***, One of the USV spectrograms with the five highest confidence scores for “pre”- and “post”-types from each of 10 rat pairs is shown; the remaining four spectrograms are provided in Extended Data Figure 3-1. The values shown in the figure represent logistic regression scores. Scores ranging from 0 to 0.5 are classified as “pre,” while those from 0.5 to 1 are classified as “post.” Additionally, values closer to 0 indicate a higher confidence in the classification as “pre,” while values closer to 1 indicate a higher confidence in the classification as “post.” Arrows indicate biting- or mastication-related sounds overlapping with USVs in the spectrogram. ***C***, Distribution of visually classified USV subtypes among high-confidence “pre”- and “post”-type representative calls. USVs were first classified as “pre” or “post” using the logistic regression model. From each rat pair, the five calls with the highest classifier confidence in each category (“pre” and “post”) were selected as representative examples and visually subtyped by the experimenter (not by DeepSqueak) according to the scheme of Wright et al. (2010). The complete set of selected calls is shown in Extended Data Figure 3-1. The percentage of each subtype was calculated separately for each rat pair and then averaged across pairs, giving equal weight to each rat pair. Data are mean ± SEM (*n* = 10 rat pairs; 5 representative calls per pair for “pre”- and “post”-type calls). This analysis was intended to illustrate the spectrographic characteristics of calls most strongly associated with “pre”- and “post”-type category and does not represent the full repertoire or relative prevalence of all USV subtypes present in the recordings. ***D***, Distribution of principal frequency for USVs classified as “pre”- and “post”-types. ***E***, Percentages of 40 kHz (30–50 kHz) flat and inverted-U types among high-confidence “pre” (score <0.25), low-confidence “pre” (0.25 < score <0.5), low-confidence “post” (0.5 < score <0.75), and high-confidence “post”-type USVs (0.75 < score). Hab1, Hab2, habituation sessions 1 and 2; Choc1, Choc2, Choc3, chocolate presentation sessions 1, 2, and 3.

**Figure 4.**
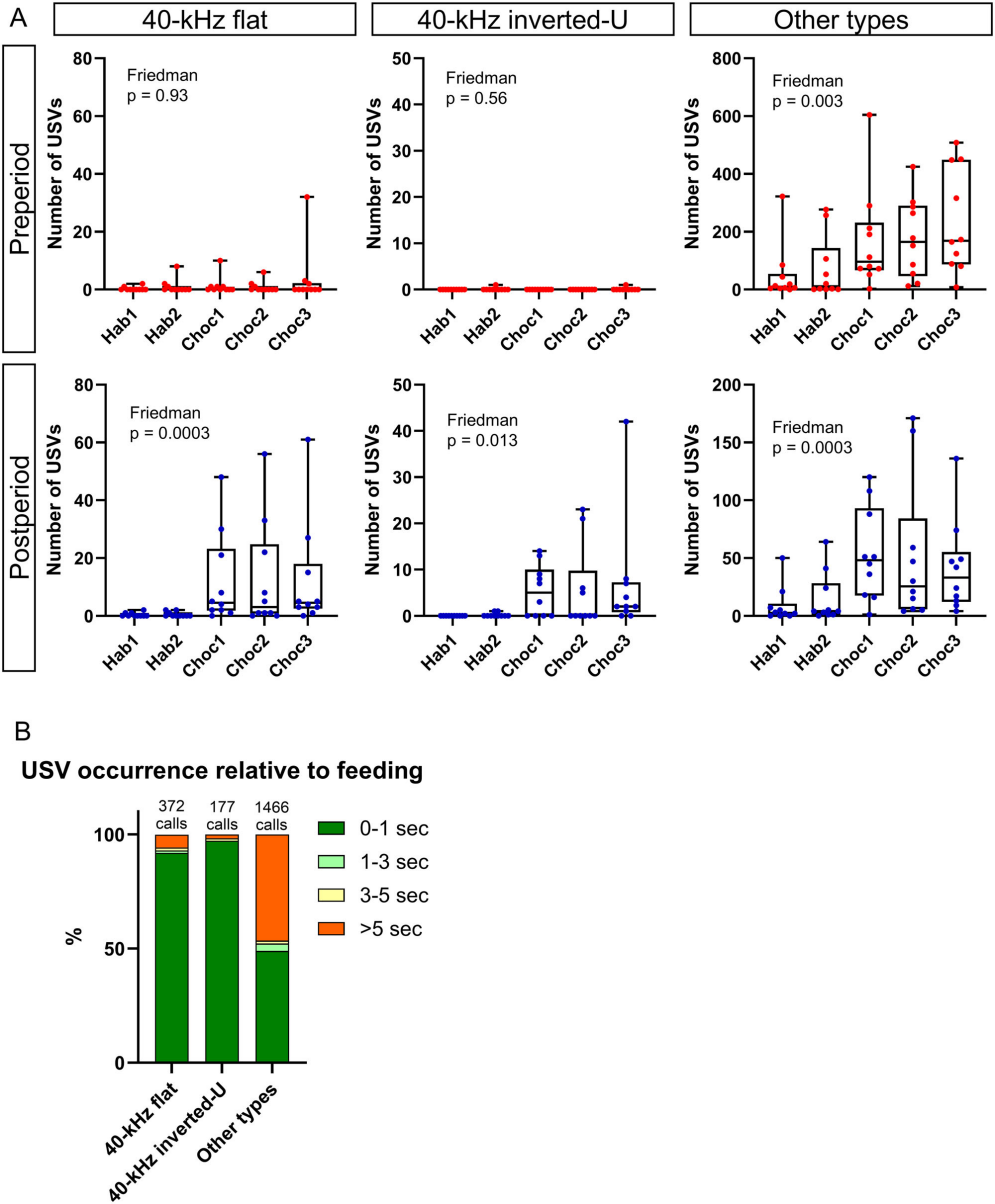
Temporal association of 40 kHz flat and inverted-U USVs with chocolate feeding. ***A***, The number of 40 kHz flat, 40 kHz inverted-U, and other types of USVs in the pre- and postperiods of each session (*n* = 10 rat pairs). ***B***, Percentages of the time intervals between 40 kHz flat, 40 kHz inverted-U, or other types of USV and the nearest instance of feeding behavior before the USV in the postperiod. Corresponding data for individual rat pair are shown in Extended Data Figure 4-1. Hab1, Hab2, habituation sessions 1 and 2; Choc1, Choc2, Choc3, chocolate presentation sessions 1, 2, and 3.

**Figure 5.**
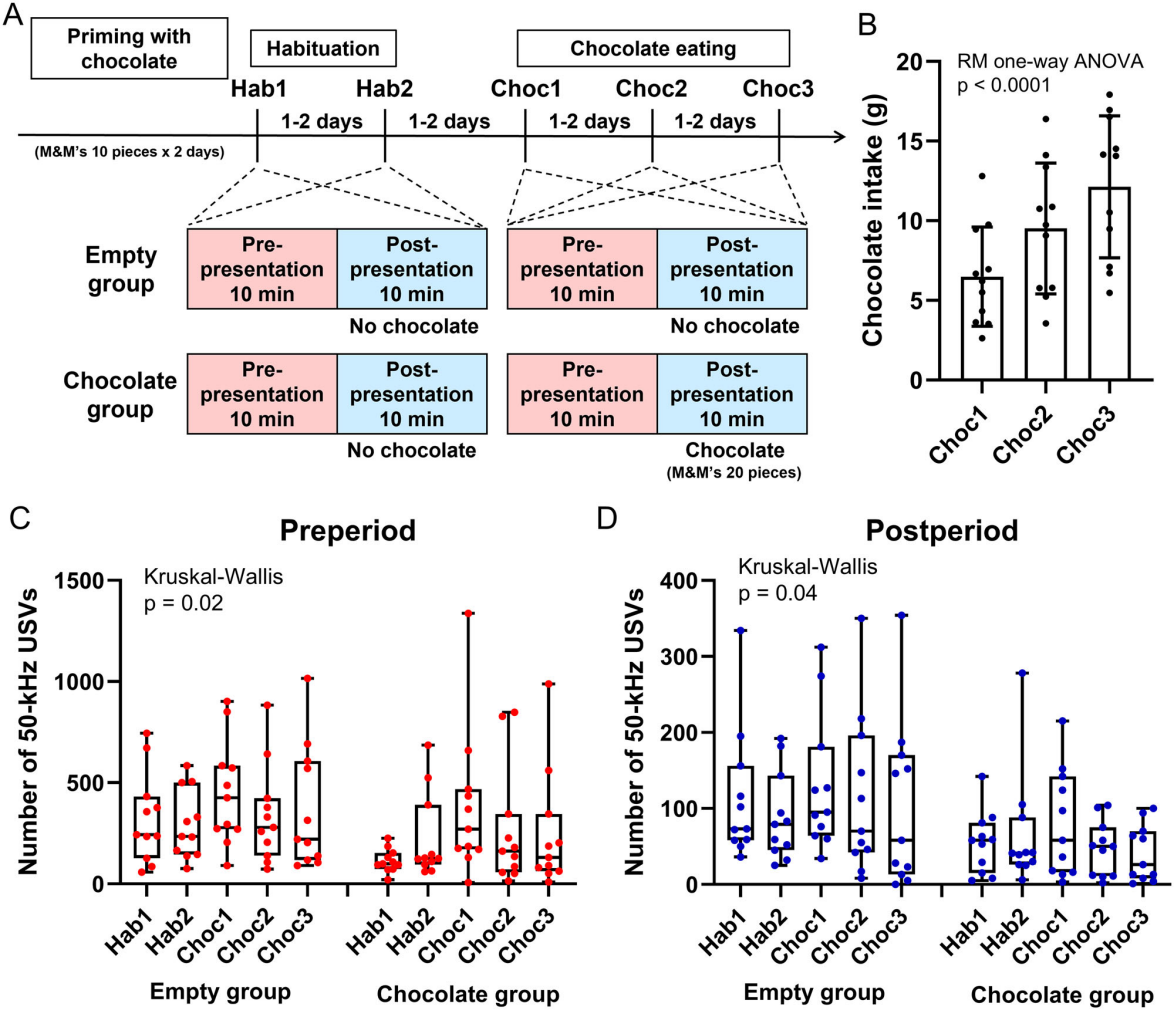
Recording of the USV test dataset. ***A***, Schedule of USV recordings to obtain test dataset for classification using the logistic regression model. The empty group served as a control and did not receive any chocolate during the pre- or postperiods. The chocolate priming phase was conducted 2–3 d before the hab1 session, during which rats were provided with 10 pieces (∼10 g) of M&M's chocolate once per day in their home cages on two occasions, with an interval of 1–2 d. Intervals between recording sessions were 1–2 d. ***B***, Amount of chocolate intake by the chocolate group during the postperiod (*n* = 11 rat pairs). ***C***, ***D***, The number of 50 kHz USVs during the pre- (***C***) and post- (***D***) periods (*n* = 11 rat pairs). Hab1, Hab2, habituation sessions 1 and 2; Choc1, Choc2, Choc3, chocolate presentation sessions 1, 2, and 3.

**Figure 6.**
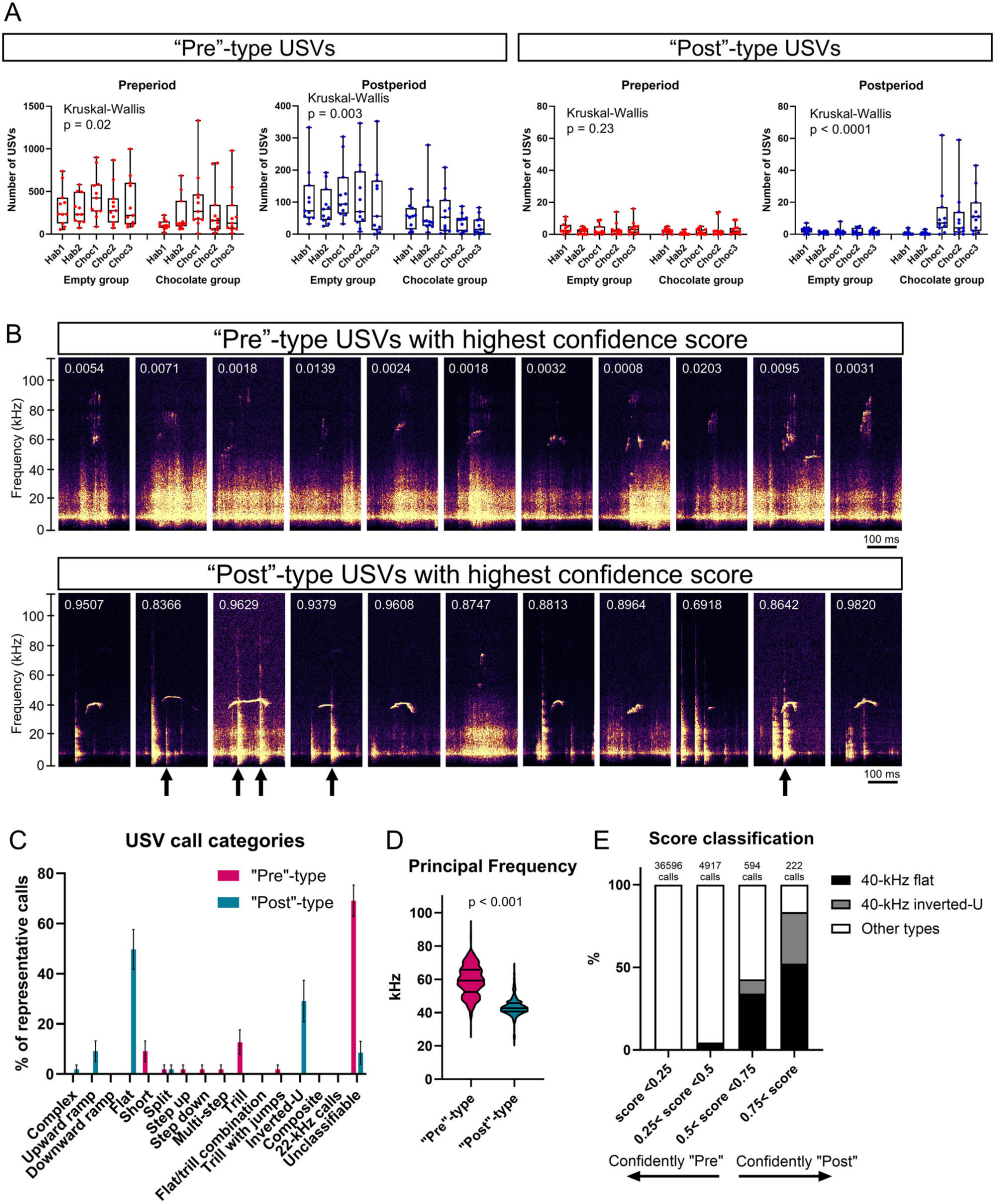
Classification of 40 kHz flat and inverted-U types of USVs as representative “post”-classified USVs in the test dataset. ***A***, The number of “pre”- and “post”-type USVs in the pre- and postperiods of each session of the empty and chocolate groups (*n* = 11 rat pairs). ***B***, One of the USV spectrograms with the five highest confidence scores for “pre”- and “post”-types from each rat pair in the chocolate group. The values shown in the figure represent logistic regression scores. Scores ranging from 0 to 0.5 are classified as “pre,” while those from 0.5 to 1 are classified as “post.” Additionally, values closer to 0 indicate a higher confidence in the classification as “pre,” while values closer to 1 indicate a higher confidence in the classification as “post.” Arrows indicate biting- or mastication-related sounds overlapping with USVs in the spectrogram. ***C***, Distribution of visually classified USV subtypes among high-confidence “pre”- and “post”-type representative calls from the chocolate group. Representative USVs were sampled on a per-pair basis using the logistic regression model. For “pre”-type calls, the five calls with the highest classifier confidence were sampled from each rat pair. For “post”-type calls, five calls were sampled where available; in two pairs with fewer “post”-type calls, all available calls (2 and 3, respectively) were included. The complete set of selected calls is shown in Extended Data Figure 6-1. The percentage of each subtype was calculated separately for each rat pair and then averaged across pairs, giving equal weight to each rat pair. Data are mean ± SEM (*n* = 11 rat pairs). ***D***, Distribution of principal frequency for USVs classified as “pre”- and “post”-types. ***E***, Percentages of 40 kHz flat (black) and inverted-U (gray) types among high-confidence “pre”, low-confidence “pre”, low-confidence “post,” and high-confidence “post”-type USVs from the entire USV test dataset. Hab1, Hab2, habituation sessions 1 and 2; Choc1, Choc2, Choc3, chocolate presentation sessions 1, 2, and 3.

**Figure 7.**
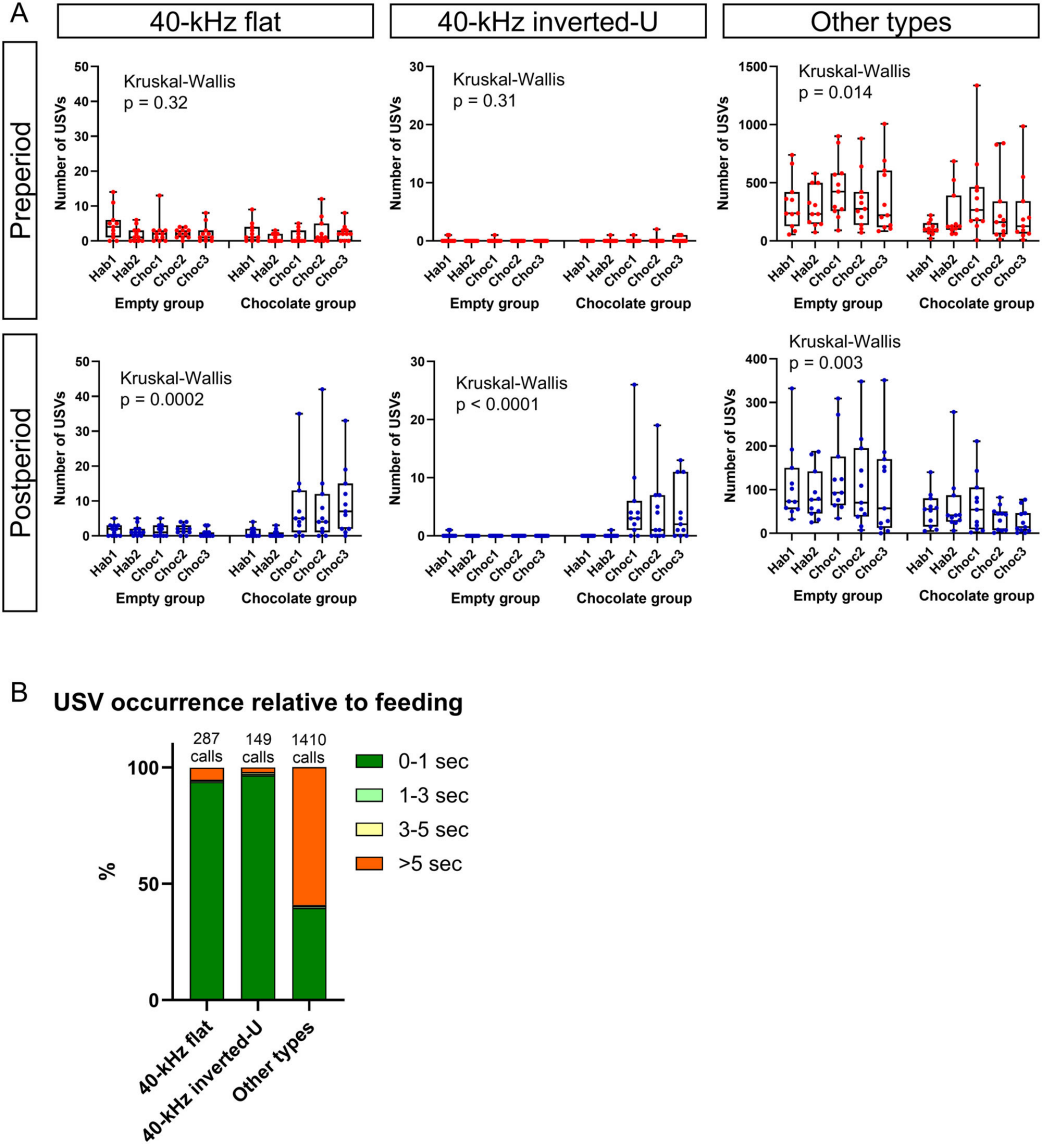
Emission of 40 kHz flat and inverted-U types of USVs during chocolate consumption in the test dataset. ***A***, The number of 40 kHz flat and inverted-U types of USVs among all USVs from 11 rat pairs in each session. ***B***, Percentages of the time intervals between 40 kHz flat, 40 kHz inverted-U, or other types of USV and the nearest instance of feeding behavior before the USV in the postperiod. Corresponding data for individual rat pair are shown in Extended Data Figure 7-1. Hab1, Hab2, habituation sessions 1 and 2; Choc1, Choc2, Choc3, chocolate presentation sessions 1, 2, and 3.

**Figure 8.**
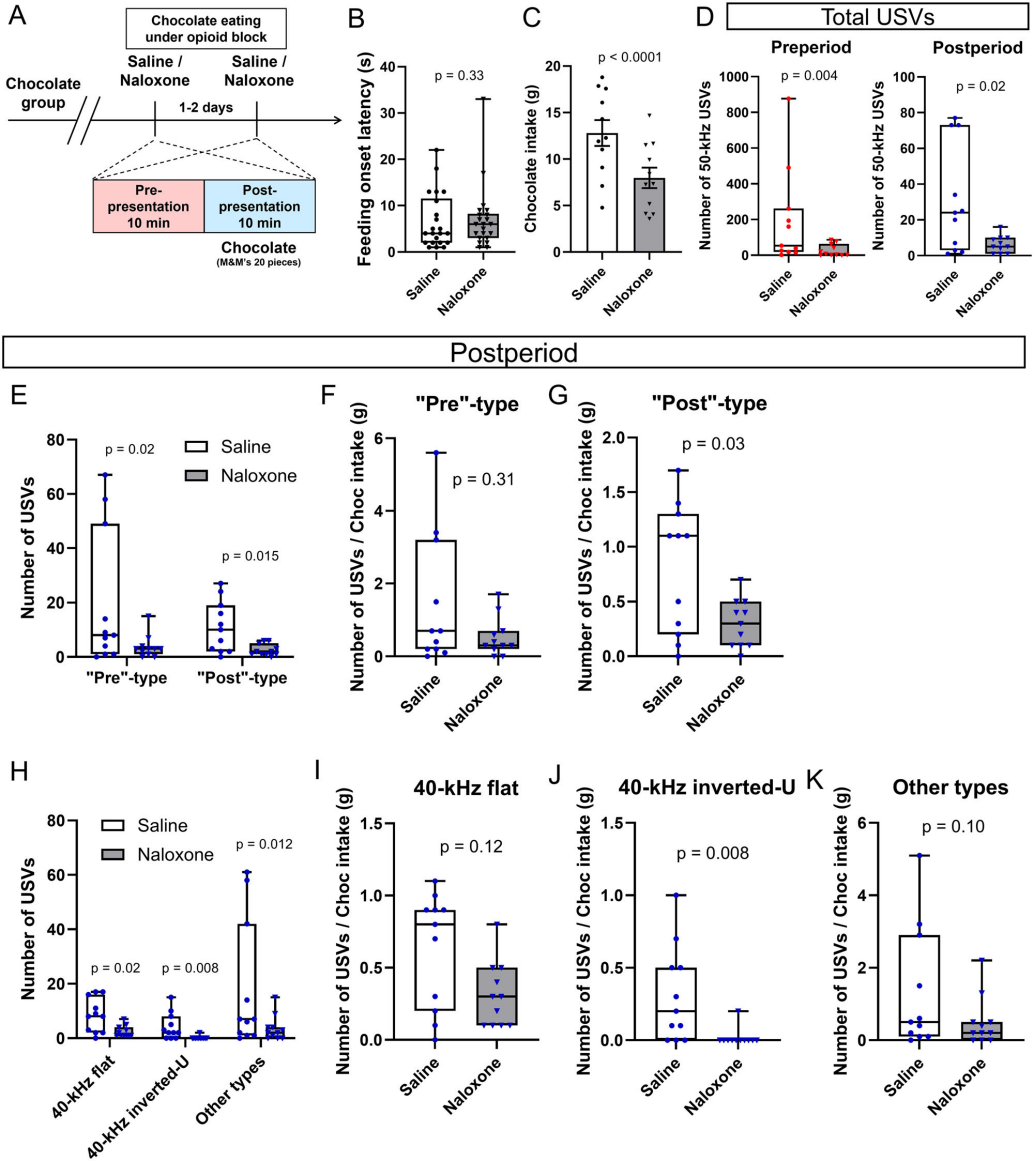
Effect of naloxone on chocolate intake and USV emission. ***A***, Schedule of USV recording after naloxone (5 mg/kg) or vehicle saline administration. Naloxone or saline was subcutaneously injected 10 min before the start of USV recording. The chocolate group used for the test dataset was further subjected to this pharmacological experiment. Each rat pair received two different injections: naloxone and saline. The order of the injections was switched among the pairs for the purpose of counterbalancing. ***B***, Feeding onset latency. ***C***, Amount of chocolate intake. ***D***, The number of total 50 kHz USVs in the preperiod (left) and postperiod (right). ***E, H***, The number of 50 kHz USVs in the postperiod. “Pre”- and “post”-type classification (***E***) and 40 kHz flat, 40 kHz inverted-U, and other-type classification (***H***). Data from the preperiod are provided in Extended Data Figure 8-1. ***F, G, I–K***, The number of USVs normalized by chocolate intake. “Pre”-type (***F***) and “post”-type (***G***) classifications and 40 kHz flat (***I***), 40 kHz inverted-U (***J***), and other-type (***K***) classification. *n* = 11 rat pairs.

First, USVs were recorded from 10 pairs of rats to create a logistic regression model which assisted visual assessment of chocolate-feeding–related USV types (training dataset; Figs. 1–4). Next, an additional set of USV recordings was collected to create a test dataset (Figs. 5–7). In this phase, 11 pairs of rats were assigned to the chocolate presentation condition (chocolate group) and subsequent pharmacological experiments (Fig. 8), while another 11 pairs served as a control group that was not exposed to chocolate (empty group). The USVs from the test dataset were classified using the logistic regression model trained on the initial datasets. This independent test dataset, including sessions without chocolate presentation, was used to evaluate whether the identified patterns using the training dataset generalized beyond time-dependent effects.

**Figure 9.**
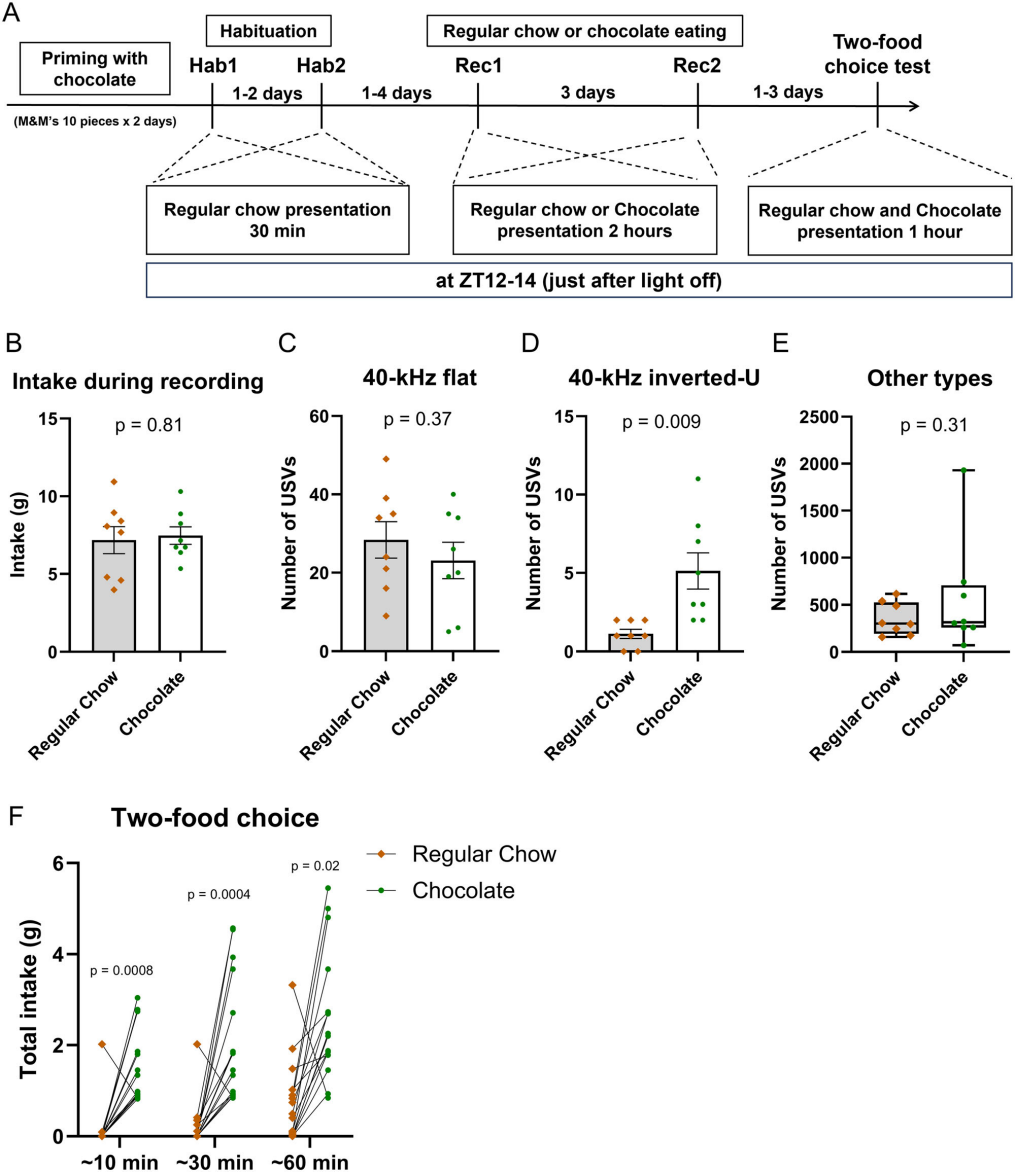
Increased occurrence of 40 kHz inverted-U USVs during chocolate feeding compared with regular chow. ***A***, Schedule of USV recording during chocolate or regular chow feeding. ***B***, Food intake during 2 h recording sessions (*n* = 8 rat pairs). ***C–E***, Number of USVs during 2 h recording sessions for 40 kHz flat (***C***), 40 kHz inverted-U (***D***), and other types of USVs (***E***; *n* = 8 rat pairs). ***F***, Food intake during a two-food choice test (*n* = 16 rats). Hab1, Hab2, habituation sessions 1 and 2; Rec1, Rec2, recording sessions 1 and 2 with regular chow or chocolate presentation.

Finally, additional eight pairs of rats were included to compare USVs emitted during chocolate feeding with those emitted during consumption of regular chow pellets (DC-8, CLEA Japan; Fig. 9). After USV recordings, each rat was individually subjected to a two-food choice test between chocolate and regular chow to assess preference between the two foods.

To optimize USV detection, experiments were conducted using pairs of rats, as previous studies have demonstrated that USV emission is more frequent in social contexts than when animals are housed individually (Inagaki et al., 2013; Wöhr and Schwarting, 2013; Brudzynski, 2015). At the same time, the group size was limited to dyads to enable future identification of individual callers while preserving the social context. In addition, only male pairs were used to avoid confounding effects of courtship or mating behavior and to avoid variability related to the estrous cycle (Matochik et al., 1992), thereby ensuring a more controlled assessment of feeding-associated vocalizations.

### USV and video recording apparatus

USVs were recorded using a USB ultrasound microphone (M500-384, frequency range: 10–160 kHz; ADC resolution, 16 bits, Pettersson Elektronik) suspended from the ceiling of a soundproof box measuring 58 × 52 × 46 cm. There were two USB cameras (C920n, Logicool Japan, and BSW305MBK, Buffalo) positioned to capture side and back views of the rat cage, along with a red LED light (LDA1R-G-G ELPA, Japan) to provide illumination. USV recordings were acquired using the BatSound Touch Lite software (Pettersson Elektronik). OBS Studio (OBS Project) was used to capture the BatSound display (including spectrograms and wave-file timestamps) simultaneously with video recordings from two USB cameras. By aligning timestamps across these recordings, this setup enabled precise temporal synchronization between USV events and behavioral video, allowing offline evaluation of animal behavior at the time of each USV emission. This setup allowed for synchronized visual inspection of both USVs and behavioral states, with minimal time discrepancy between the two, estimated to be within 1–2 video frames (≤66 ms at 30 fps).

### Recording of training dataset

Figure 1*A* shows the recording schedule for the training dataset, which consisted of three parts: habituation sessions, where USVs were recorded in the soundproof box without chocolate presentation (two sessions, hab1 and hab2); chocolate priming, during which rats were provided with four pieces of M&M's milk chocolate (Mars, ∼4 g in total, corresponding to 0.9–1.0 g per piece) per day in their home cages for 3 consecutive days with no USV recording; and chocolate presentation sessions (three sessions, choc1, choc2, and choc3). Chocolate priming was implemented to reduce neophobia toward chocolate.

Twenty minutes before the start of the recording, the bedding of the rat cages was replaced, pellet food was removed from the cage lid, and the rats were left in their home environment. Then, a pair of rats was transferred to the soundproof box in their home cage. The USVs and behaviors exhibited by the rats were recorded for the first 10 min (prechocolate presentation period). Then, the experimenter opened the soundproof box door and the cage lid and placed a hand into the cage (for habituation sessions) or a glass jar containing M&M's milk chocolate (20 pieces, ∼18–20 g in total) into the cage. The lid and door were then closed, and further recording of USVs and behaviors took place for an additional 10 min (postchocolate presentation period). After each recording session, the rats were returned to their home environment and provided with pellet food.

### Recording of test dataset

As shown in Figure 5*A*, we initially conducted chocolate priming before starting USV recording, aiming to increase USV emissions during both the habituation (hab1 and hab2) and chocolate presentation (choc1, choc2, and choc3) sessions. During priming, rats were provided with 10 pieces (∼9–10 g in total) of M&M's milk chocolate per day in their home cages on two occasions with an interval of 1–2 d. We then proceeded to perform two habituation sessions, recording USV calls in the soundproof box without chocolate presentation (hab1 and hab2), followed by three chocolate presentation sessions (choc1, choc2, and choc3). The recording procedure was the same as for the training dataset, except that the empty group received an empty glass jar during the postchocolate presentation period in the choc1, choc2, and choc3 sessions. After the choc3 session, the chocolate group underwent pharmacological experiments using naloxone (Fig. 8).

Rat pairs assigned to the chocolate and empty control conditions were tested in parallel within the same experimental batch. Sessions for both groups were conducted during the same experimental period using identical procedures, apparatus, and recording parameters, with the presence or absence of chocolate during the postperiod as the only experimental manipulation.

### Pharmacological experiments

To investigate the involvement of the opioid system in USV emission, we used naloxone, a nonselective competitive opioid receptor antagonist with a higher affinity for μ-opioid receptors than for κ- and δ-opioid receptors. Naloxone hydrochloride salt (144-09411, FUJIFILM Wako Pure Chemical) was dissolved in a saline solution at a concentration of 5 mg/ml. The rats received a subcutaneous injection of either naloxone or saline at a volume of 1 ml/kg body weight, administered 10 min prior to the start of USV recordings. The injection order (naloxone or saline) was alternated between pairs of rats, with six pairs receiving naloxone first, followed by saline, and five pairs receiving saline first, followed by naloxone. The recording procedure was the same as that used for the chocolate presentation sessions.

### Recording of regular chow-feeding dataset and two-food choice test

Figure 9*A* shows the recording schedule for the regular chow-feeding dataset and the subsequent two-food choice tests. We initially conducted chocolate priming before starting USV recording by providing rats with 10 pieces (∼9–10 g in total) of M&M's milk chocolate per day in their home cages on two occasions with an interval of 1–2 d. We then conducted two habituation sessions (hab1 and hab2), during which rats were placed in a soundproof box while regular chow was presented for 30 min. This was followed by two sessions (rec1 and rec2), in which either chocolate or regular chow (40 g) was freely available for 2 h.

After the recording sessions, rats were subjected to a two-food choice test to assess their preference between chocolate and regular chow. During the tests, rat pairs were separated and housed individually in adjacent cages within the same row of the home rack. Two glass jars containing either 20 g of chocolate or 20 g regular chow were placed in the corners of each cage. Rats were allowed to access the food for 60 min, and food intake was measured at 10, 30, and 60 min after presentation.

### USV detection by DeepSqueak with manual validation

We used DeepSqueak v3.0.1 with MATLAB 2022b to generate candidate 50 kHz USV events (Coffey et al., 2019) and to export acoustic parameters. The Rat Detector YOLO R1.mat model for the DeepSqueak platform was utilized for USV detection, with a cutoff range of 18–100 kHz. Following the automated detection, all detected events were subsequently reviewed by trained human raters using spectrogram inspection and audio playback (including slow playback when necessary) to exclude nonvocal artifacts (e.g., broadband signals associated with rat locomotion typically centered at ∼5 kHz, as well as water-intake sounds). We also visually detected 22-kHz-long USVs and excluded them from the dataset. To assess the detection performance of DeepSqueak for trill calls (Wright et al., 2010), which are reported to be challenging for automated contour-based classification, we performed visual verification of trill calls by human raters across all chocolate consumption sessions (choc1, choc2, and choc3) of the 10 rat pairs in the training dataset. Trill calls were identified according to the criteria of Wright et al. (2010), defined as 50 kHz USVs containing multiple cycles of regular frequency modulation in the 30–80 kHz range without abrupt frequency jumps or extended flat components. In the present inspection, we required at least three cycles of frequency modulation for a call to be classified as a trill. Across the 30 inspected sessions, we manually identified 443 trill calls, of which 439 (99.1%) were successfully detected by DeepSqueak.

DeepSqueak provides 11 parameters for the identified USVs, including call length, principal frequency, low frequency, high frequency, delta frequency, standard deviation of frequency, slope, sinuosity, mean power, tonality, and peak frequency. Tonality is defined as the degree to which acoustic energy is concentrated around a dominant frequency relative to broadband noise. These parameters were exported to CSV files for the training of a logistic regression model and further classification of USVs.

### Supervised machine learning classification of USVs using logistic regression

We created a logistic regression model to classify USVs using the Classification Learner application in MATLAB 2022b. For the training dataset, all USVs detected by DeepSqueak during the three sessions in which chocolate was presented to 10 pairs of rats (choc1, choc2, and choc3) were used (Figs. 1, 2). USVs emitted before the chocolate presentation (preperiod) were labeled as “pre,” whereas those emitted after the chocolate presentation (postperiod) were labeled as “post.” A total of 7,909 calls (5,893 preperiod and 2,016 postperiod calls) were used for training, providing sufficient statistical power to detect systematic differences in acoustic features between conditions (Table 1). The model classified the USVs into “pre”-type and “post”-type based on 11 generated parameters (Table 2) and was subsequently applied to classify both the training and test datasets.

10.1523/ENEURO.0356-25.2026.f2-1 Figure 2-1 **Pair-level comparison of acoustic parameters.** This figure shows a pair-level breakdown of the pooled data presented in Figs. 2A–K. Each dot represents the median value for a single rat pair. Download Figure 2-1, TIF file.

**Table 1.** AUC and accuracy of our logistic regression model

	AUC	Accuracy
Our model	0.78	0.83
Model with data shuffling	0.51	0.83

**Table 2.** Coefficients of our logistic regression model

Call length	24
Principal frequency	0.014
Low frequency	0
High frequency	−0.12
Delta frequency	−0.0067
Frequency standard deviation	0.0059
Slope	0.00021
Sinuosity	0.010
Mean power	−0.16
Tonality	14
Peak frequency	0.0039

Although the classifier labels are referred to as “pre” and “post” based on the structure of the training data, these labels do not simply reflect the temporal position of a call within a session. Instead, they denote whether a call was emitted under chocolate-available or chocolate-absent conditions, as validated using sessions without chocolate and an independent test dataset.

### Visual classification of USV subtypes

As shown in Figures 3*C* and 6*C*, USVs classified as “pre”-type or “post”-type by the logistic regression model were visually reclassified by the experimenters (not by DeepSqueak) based on conventional USV-type classification using spectrogram features into 14 categories: Complex, Upward ramp, Downward ramp, Flat, Short, Split, Step up, Step down, Multi-step, Trill, Flat/trill, Trill with jumps, Inverted-U, and Composite (Wright et al., 2010). USVs that did not fall into any of these categories were labeled as “unclassifiable.” For each pair of rats in the training dataset (10 pairs) and the chocolate group in the test dataset (11 pairs), the top five USVs with the highest confidence scores for being either “pre”-type or “post”-type were chosen for visual classification as illustrative examples (Figs. 3*B*, 6*B*; Extended Data Figs. 3-1, 6-1); this subset was not intended to represent the complete subtype distribution in each condition. In these representative subtype analyses, calls were sampled on a per-pair basis with approximately balanced contributions across pairs. When fewer calls were available for a given category, all available calls were included.

10.1523/ENEURO.0356-25.2026.f3-1 Figure 3-1 **Representative ‘pre’- and ‘post’-type USVs of the training dataset.** Five USVs with the highest confidence score from each of the 10 pair for classification as ‘pre’- or ‘post’-type were selected. The values shown in the figure represent logistic regression scores. Scores ranging from 0 to 0.5 are classified as ‘pre,’ while those from 0.5 to 1 are classified as ‘post.’ Additionally, values closer to 0 indicate a higher confidence in the classification as ‘pre,’ while values closer to 1 indicate a higher confidence in the classification as ‘post.’ Download Figure 3-1, TIF file.

10.1523/ENEURO.0356-25.2026.f4-1 Figure 4-1 **Pair-wise latency distributions of USVs relative to feeding onset in the training dataset**. Data are shown separately for (A) 40-kHz flat, (B) 40-kHz inverted-U, and (C) other USV types. Colors indicate delay bins (0-1, 1-3, 3-5, and >5 sec). Download Figure 4-1, TIF file.

10.1523/ENEURO.0356-25.2026.f6-1 Figure 6-1 **Representative ‘pre’- and ‘post’-type USVs of the test dataset.** Five USVs with the highest confidence score from each of the 11 pair in the chocolate group for classification as ‘pre’- or ‘post’-type were selected. For Pairs 12 and 19, fewer than five ‘post’-type USVs were available. The values shown in the figure represent logistic regression scores. Scores ranging from 0 to 0.5 are classified as ‘pre,’ while those from 0.5 to 1 are classified as ‘post.’ Additionally, values closer to 0 indicate a higher confidence in the classification as ‘pre,’ while values closer to 1 indicate a higher confidence in the classification as ‘post.’ Download Figure 6-1, TIF file.

10.1523/ENEURO.0356-25.2026.f7-1 Figure 7-1 **Pair-wise latency distributions of USVs relative to feeding onset in the test dataset**. Data are shown separately for (A) 40-kHz flat, (B) 40-kHz inverted-U, and (C) other USV types. Colors indicate delay bins (0-1, 1-3, 3-5, and >5 sec). Download Figure 7-1, TIF file.

10.1523/ENEURO.0356-25.2026.f8-1 Figure 8-1 **Effect of naloxone on USV emission.** A, B: Number of 50-kHz USVs in the pre-period; (A) ‘pre’- and ‘post’-type classification and (B) 40-kHz flat, 40-kHz inverted-U, and other type classification. Download Figure 8-1, TIF file.

We then visually examined all USVs from both the training and test datasets and classified them into three types: 40 kHz flat type (near-constant frequency with a mean slope between −0.2 and 0.2 kHz/ms, with a principal frequency range of 30–50 kHz), 40 kHz inverted-U type (a monotonic increase followed by a monotonic frequency decrease, each of at least 5 kHz, with a principal frequency range of 30–50 kHz), or other types. These categories were defined in a data-driven manner based on subtype features that emerged as prominent in the comparison between “pre”- and “post”-type USVs (see Results).

### Visual analysis of feeding behavior

Feeding behaviors associated with USV occurrence during the postperiod of chocolate presentation sessions (choc1, choc2, and choc3) were assessed visually by the experimenter using USB camera recordings. For each USV, the time interval (in seconds) from the most recent instance in which either or both rats exhibited chocolate biting or mastication behavior to the occurrence of that USV was measured. The intervals were then classified into four categories: 0–1 s, 1–3 s, 3–5 s, and >5 s (Figs. 4*B*, 7*B*; Extended Data Figs. 4-1, 7-1).

In the naloxone administration study (Fig. 8), latency from chocolate presentation to the first oral contact was quantified from video recordings to assess potential nonspecific effects of naloxone on general mobility and food-directed behavior.

### Statistical analyses

For datasets subjected to statistical analysis, normality was initially assessed using the Shapiro–Wilk test. When all datasets followed a normal distribution, data were presented as means with standard deviations using bar charts, and parametric tests were applied. If any dataset deviated from normality, data were presented as medians with interquartile ranges using box-and-whisker plots or violin plots, and nonparametric tests were used. Repeated-measures ANOVA was performed without assuming sphericity, and the Greenhouse–Geisser correction was applied to adjust the degrees of freedom. All statistical analyses were conducted using GraphPad Prism 10. The number of replicates was determined by referencing similar previous studies and is specified for each experiment in the Results section. The outcomes of the statistical tests are summarized in Tables 3–5.

**Table 3.** Summary of statistical analyses, Part 1 (Figs. 1–3)

Figure	Description	Analysis	Test statistics	*p* value	Sample size
1*B*	Chocolate intake	Repeated-measures one–way ANOVA	*F*_(1.93,17.4)_ = 8.91	*p* = 0.002	*n* = 10 pairs per group
1*C*	Number of USVs in preperiod	Friedman test	*χ*²_(4)_ = 11.96	*p* = 0.02	*n* = 10 pairs per group
1*D*	Number of USVs in postperiod	Friedman test	χ²_(4)_ = 22.31	*p* = 0.0002	*n* = 10 pairs per group
2*A*	Call length	Mann–Whitney	*U* = 4.5 × 10^6^	*p* < 0.001	*n* = 5,893 calls (pre), 2016 calls (post)
2*B*	Principal frequency	Mann–Whitney	*U* = 3.2 × 10^6^	*p* < 0.001	*n* = 5,893 calls (pre), 2016 calls (post)
2*C*	Low frequency	Mann–Whitney	*U* = 3.4 × 10^6^	*p* < 0.001	*n* = 5,893 calls (pre), 2016 calls (post)
2*D*	High frequency	Mann–Whitney	*U* = 2.9 × 10^6^	*p* < 0.001	*n* = 5,893 calls (pre), 2016 calls (post)
2*E*	Delta frequency	Mann–Whitney	*U* = 5.1 × 10^6^	*p* < 0.001	*n* = 5,893 calls (pre), 2016 calls (post)
2*F*	Frequency standard deviation	Mann–Whitney	*U* = 4.9 × 10^6^	*p* < 0.001	*n* = 5,893 calls (pre), 2016 calls (post).
2*G*	Slope	Mann–Whitney	*U* = 5.0 × 10^6^	*p* < 0.001	*n* = 5,893 calls (pre), 2016 calls (post)
2*H*	Sinuosity	Mann–Whitney	*U* = 5.3 × 10^6^	*p* < 0.001	*n* = 5,893 calls (pre), 2016 calls (post)
2*I*	Mean power	Mann–Whitney	*U* = 4.0 × 10^6^	*p* < 0.001	*n* = 5,893 calls (pre), 2016 calls (post)
2*J*	Tonality	Mann–Whitney	*U* = 3.3 × 10^6^	*p* < 0.001	*n* = 5,893 calls (pre), 2016 calls (post)
2*K*	Peak frequency	Mann–Whitney	*U* = 3.2 × 10^6^	*p* < 0.001	*n* = 5,893 calls (pre), 2016 calls (post)
3*A*	“Pre”-type USVs in preperiod “Pre”-type USVs in postperiod “Post”-type USVs in preperiod “Post”-type USVs in postperiod	Friedman Friedman Friedman Friedman	χ²_(4)_ = 18.62 χ²_(4)_ = 22.76 χ²_(4)_ = 1.27 χ²_(4)_ = 19.73	*p* = 0.002 *p* = 0.0001 *p* = 0.87 *p* = 0.0006	*n* = 10 pairs per group *n* = 10 pairs per group *n* = 10 pairs per group *n* = 10 pairs per group

**Table 4.** Summary of statistical analyses, Part 2 (Figs. 4–7)

Figure	Description	Analysis	Test statistics	*p* value	Sample size
4*A*	40 kHz flat USVs in preperiod 40 kHz flat USVs in postperiod 40 kHz inverted-U USVs in preperiod 40 kHz inverted-U USVs in postperiod Other types of USVs in preperiod Other types of USVs in postperiod	Friedman Friedman Friedman Friedman Friedman Friedman	χ²_(4)_ = 0.89 χ²_(4)_ = 21.51 χ²_(4)_ = 3.00 χ²_(4)_ = 12.65 χ²_(4)_ = 15.76 χ²_(4)_ = 20.81	*p* = 0.93 *p* = 0.0003 *p* = 0.56 *p* = 0.013 *p* = 0.003 *p* = 0.0003	*n* = 10 pairs per group *n* = 10 pairs per group *n* = 10 pairs per group *n* = 10 pairs per group *n* = 10 pairs per group *n* = 10 pairs per group
5*B*	Chocolate intake	Repeated-measures one–way ANOVA	*F*_(1.85,18.5)_ = 51.29	*p* < 0.0001	*n* = 11 pairs per group
5*C*	Number of USVs in preperiod	Kruskal–Wallis	*H* = 20.32	*p* = 0.02	*n* = 11 pairs per group
5*D*	Number of USVs in postperiod	Kruskal–Wallis	*H* = 17.34	*p* = 0.04	*n* = 11 pairs per group
6*A*	“Pre”-type USVs in preperiod “Pre”-type USVs in postperiod “Post”-type USVs in preperiod “Post”-type USVs in postperiod	Kruskal–Wallis Kruskal–Wallis Kruskal–Wallis Kruskal–Wallis	*H* = 20.36 *H* = 24.60 *H* = 11.71 *H* = 36.72	*p* = 0.02 *p* = 0.003 *p* = 0.23 *p* < 0.0001	*n* = 11 pairs per group *n* =11 pairs per group *n* = 11 pairs per group *n* = 11 pairs per group
7*A*	40 kHz flat USVs in preperiod 40 kHz flat USVs in postperiod 40 kHz inverted-U USVs in preperiod 40 kHz inverted-U USVs in postperiod Other types of USVs in preperiod Other types of USVs in postperiod	Kruskal–Wallis Kruskal–Wallis Kruskal–Wallis Kruskal–Wallis Kruskal–Wallis Kruskal–Wallis	*H* = 10.35 *H* = 31.54 *H* = 10.50 *H* = 63.01 *H* = 20.64 *H* = 25.17	*p* = 0.32 *p* = 0.0002 *p* = 0.31 *p* < 0.0001 *p* = 0.014 *p* = 0.003	*n* = 11 pairs per group *n* = 11 pairs per group *n* = 11 pairs per group *n* = 11 pairs per group *n* = 11 pairs per group *n* = 11 pairs per group

**Table 5.** Summary of statistical analyses, Part 3 (Figs. 8, 9)

Figure	Description	Analysis	Test statistics	*p* value	Sample size
8*B*	Feeding onset latency	Wilcoxon	*W* = 53.00	*p* = 0.33	*n* = 22 rats per group
8*C*	Chocolate intake	Paired *t* test	*t*_(10)_ = 6.93	*p* < 0.0001	*n* = 11 pairs per group
8*D*	Total USVs in preperiod Total USVs in postperiod	Wilcoxon Wilcoxon	*W* = −53.00 *W* = −52.00	*p* = 0.004 *p* = 0.02	*n* = 11 pairs per group *n* = 11 pairs per group
8*E*	“Pre”-type USVs “Post”-type USVs	Wilcoxon Wilcoxon	*W* = −34.00 *W* = −53.00	*p* = 0.02 *p* = 0.015	*n* = 11 pairs per group *n* = 11 pairs per group
8*F*	Number of USVs/Choc intake	Wilcoxon	*W* = −18.00	*p* = 0.31	*n* = 11 pairs per group
8*G*	Number of USVs/Choc intake	Wilcoxon	*W* = −43.00	*p* = 0.03	*n* = 11 pairs per group
8*H*	40 kHz flat USVs 40 kHz inverted-U USVs Other types of USVs	Wilcoxon Wilcoxon Wilcoxon	*W* = −52.00 *W* = −36.00 *W* = −42.00	*p* = 0.02 *p* = 0.008 *p* = 0.012	*n* = 11 pairs per group *n* = 11 pairs per group *n* = 11 pairs per group
8*I*	Number of USVs/Choc intake	Wilcoxon	*W* = −27.00	*p* = 0.12	*n* = 11 pairs per group
8*J*	Number of USVs/Choc intake	Wilcoxon	*W* = −36.00	*p* = 0.008	*n* = 11 pairs per group
8*K*	Number of USVs/Choc intake	Wilcoxon	*W* = −29.00	*p* = 0.10	*n* =11 pairs per group
9*B*	Intake	Paired *t* test	*t*_(7)_ = 0.25	*p* = 0.81	*n* = 8 pairs per group
9*C*	Number of USVs	Paired *t* test	*t*_(7)_ = 0.96	*p* = 0.37	*n* = 8 pairs per group
9*D*	Number of USVs	Paired *t* test	*t*_(7)_ = 3.58	*p* = 0.009	*n* = 8 pairs per group
9*E*	Number of USVs	Wilcoxon	*W* = 16.00	*p* = 0.31	*n* = 8 pairs per group
9*F*	Total intake for 10 min Total intake for 30 min Total intake for 60 min	Wilcoxon Wilcoxon Wilcoxon	*W* = 120.00 *W* = 124.00 *W* = 114.00	*p* = 0.0008 *p* = 0.0004 *p* = 0.02	*n* = 16 rats per group *n* = 16 rats per group *n* = 16 rats per group

### Data availability statement

The data that support the findings of this study, including USV audio files (WAV format), behavioral video files (MP4 format), USV detection files generated by DeepSqueak (MAT format), and statistical analysis files, are available from the corresponding author, Koshi Murata, upon reasonable request. The logistic regression model will be shared via an online repository after acceptance.

## Results

### Increased emission of 50 kHz USVs during both pre- and postchocolate presentation periods

To investigate whether rats emit specific subtypes of USVs when consuming palatable foods, we recorded USVs during chocolate feeding. Adult male Sprague Dawley rats were housed in pairs and placed in a soundproof box for USV recording, where they were allowed access to M&M's milk chocolate. Each recording session consisted of a 10 min prepresentation period (preperiod), during which the rats did not have access to the chocolate, followed by a 10 min postpresentation period (postperiod). To habituate the rats to the soundproof box and establish baseline vocalization levels, two habituation sessions (hab1 and hab2) were carried out before chocolate presentation. During these sessions, the door of the soundproof box and the lid of the cage were opened at the end of the preperiod to simulate chocolate delivery and habituate the rats to the experimenter's hand intrusion without presenting any chocolate. After the habituation sessions, the rats were primed with four pieces of M&M's chocolate (∼4 g per day for 3 consecutive days) in their home environment. Following this, three sessions of chocolate presentation (choc1, choc2, and choc3) were carried out. During these sessions, the home cages containing each rat pair were placed in the soundproof box, and their USVs were recorded for 10 min (preperiod). Subsequently, 20 pieces of chocolate (∼20 g) were provided to the rats, and their USVs were recorded for another 10 min (postperiod). An interval of 1–2 d was maintained between the recording sessions (Fig. 1*A*). Rat behaviors were video-recorded, and chocolate intake was measured. Using DeepSqueak, we identified 50 kHz (30–100 kHz) USVs from the recorded audio data. Notably, fasting was not part of the experimental protocol.

Chocolate consumption increased slightly across the three chocolate presentation sessions (repeated-measures one–way ANOVA, *F*_(1.93,17.4)_ = 8.91; *p* = 0.002; Fig. 1*B*). Although rats did not consume all available chocolate during the recording period, the amount consumed (∼1–2% of body weight within 10 min) represents substantial intake under nonfasting conditions. During the preperiod of the two habituation sessions, most rat pairs emitted a low number of 50 kHz USVs (Fig. 1*C*). In contrast, there was a significant increase in USVs during the preperiod of the three sessions that included chocolate presentation (Friedman test, *p* = 0.02; Fig. 1*C*). Similarly, during the postperiod of the two habituation sessions, most rats emitted few USV calls, but a significant increase in USV calls was observed during those sessions that included chocolate presentation (Friedman test, *p* = 0.0002; Fig. 1*D*).

### Distinct USV spectrograms between pre- and postchocolate presentation periods

We then proceeded to examine whether the spectrograms of the 50 kHz USVs emitted during the postperiod when chocolate was available differed from those emitted during the preperiod (when chocolate was unavailable). We conducted a statistical analysis that compared 11 parameters (call length, principal frequency, low frequency, high frequency, delta frequency, standard deviation of frequency, slope, sinuosity, mean power, tonality, and peak frequency) of the USV spectrograms measured by DeepSqueak between the pre- and postperiods of three sessions in which chocolate was presented. There was a total of 5,893 USV calls in the preperiod and 2,016 USV calls in the postperiod from sessions choc1, choc2, and choc3. All 11 parameters exhibited significant differences between the pre- and postperiods, as determined by the Mann–Whitney *U* test (Fig. 2*A*–*K*). USVs emitted during the postperiod were characterized by lower frequency, longer duration, increased loudness, and improved signal-to-noise ratio compared with those emitted during the preperiod. Because multiple calls were obtained from each rat pair, we additionally performed pair-level analyses in which median values were calculated for each pair and compared between pre- and postperiods, to provide an adequately conservative statistical assessment (Extended Data Fig. 2-1). When analyses were repeated using pair-level median values, although not all acoustic parameters remained significant, the call length, principal frequency-related measures, call length, mean power, and tonality showed consistent and robust effects.

To obtain representative examples of USV spectrograms from both the pre- and postperiods, we selected five USVs that were closest to the median values of the 11 parameters in each period. The USVs from the preperiod showed various shapes of spectrograms in the 50–60 kHz range. In contrast, the USVs from the postperiod exhibited inverted-U, flat, and upward ramp shapes in the 40–50 kHz range (Fig. 2*L*).

### Classification of “post”-type USVs using a supervised machine learning model, highlighting 40 kHz flat and 40 kHz inverted-U types of USVs during chocolate feeding

To identify USV subtypes unique to the postperiod, we then examined if a supervised machine learning model could distinguish between USVs as “pre”-type or “post”-type by analyzing differences in the parameters of USV spectrograms between the pre- and postperiods. A logistic regression model was developed using the 11 parameters presented in Figure 2*A*–*K* to categorize USVs as either “pre”-type or “post”-type. The training dataset included USVs from three chocolate presentation sessions, comprising all calls in the preperiod (*n* = 5,893 calls) labeled as “preperiod type” and all calls in the postperiod (*n* = 2,016 calls) as “postperiod type.” The resulting model achieved an area under the curve (AUC) of 0.78 with an accuracy of 82.5% (Table 1). The model primarily emphasized call length and tonality (Table 2). Tonality reflects spectral concentration (see Materials and Methods) and may also be influenced by behavioral context, including locomotion-related sound.

Another model was constructed using a dataset with randomized preperiod and postperiod labels, which yielded an AUC at the chance level (0.51), classifying all USVs as “pre”-type. The higher AUC in the original model suggests that USVs in the postperiod have distinct spectrograms compared with those in the preperiod and that our model effectively used these differences to classify USVs into the “pre”- and “post”-types.

We applied the classifier model to the USVs emitted during both the two habituation sessions and the three chocolate presentation sessions, which were utilized as training datasets. The number of USVs classified as “pre”- and “post”-types were assessed (Fig. 3*A*). “Pre”-type USVs were emitted in both the pre- and postperiods across all sessions. In both periods, the number of “pre”-type calls showed a tendency to increase in the chocolate presentation sessions. “Post”-type USVs, in contrast, were rare during the preperiod in all sessions, with no significant increase observed across the chocolate sessions. During the postperiod, however, “post”-type calls remained scarce in the habituation sessions but increased to significant levels during the chocolate sessions. These results suggest that “post”-type USVs were seldom emitted in the absence of chocolate but became more prevalent when chocolate was available to the rats.

Next, we analyzed the spectrogram of USVs classified as “pre”-type and “post”-type. From each pair of rats, we selected the five USVs with the highest classification confidence, as representative examples (Fig. 3*B*; Extended Data Fig. 3-1), and evaluated them visually based on conventional USV-type categories (Wright et al., 2010). The “pre”-type USVs with high confidence scores were mainly short or unclassifiable. Conversely, most of the “post”-type USVs with high confidence scores belonged to the flat or inverted-U types, accounting for 62 and 18%, respectively (Fig. 3*C*). Besides the spectrogram patterns, “post”-type USVs were characterized by a lower-frequency range than “pre”-type USVs, which primarily ranged ∼40 kHz (30–50 kHz; Fig. 3*D*). Both flat and inverted-U types exhibited a principal frequency range within 40 kHz (Fig. 3*B*), which has been reported in previous studies conducted during the consumption of a standard laboratory diet (Takahashi et al., 2010; Champeil-Potokar et al., 2023). This visual subtyping revealed that flat and inverted-U calls accounted for the majority of high-confidence “post”-type calls, prompting us to focus on these two subtypes as candidate vocal correlates of chocolate consumption.

Next, we evaluated the proportion of 40 kHz flat and 40 kHz inverted-U type USVs within those classified as “pre”- and “post”-type by the model (Fig. 3*E*). The “pre”-type USVs contained few 40 kHz flat or 40 kHz inverted-U types. In contrast, among the USVs classified as “post”-type with high confidence (logistic regression score, >0.75), 55% were 40 kHz flat USVs, and 21% were 40 kHz inverted-U USVs. Among the USVs classified as “post”-type with low confidence (logistic regression score: between 0.5 and 0.75), 19% were 40 kHz flat USVs, and 10% were 40 kHz inverted-U USVs. Since the “post”-type USVs occurred when chocolate was presented to the rats, we hypothesized that 40 kHz flat and 40 kHz inverted-U USVs were emitted when chocolate was available. We visually assessed all USVs to determine whether they were 40 kHz flat or 40 kHz inverted-U types (Fig. 4*A*). Both 40 kHz flat and 40 kHz inverted-U USVs were rarely observed when chocolate was unavailable and were instead emitted during the postperiods of the chocolate presentation sessions (choc1, choc2, and choc3).

We further investigated whether the 40 kHz flat and inverted-U USVs were associated with actual chocolate consumption. Using video recordings, we analyzed the behaviors of the rats and measured the time intervals between each USV and the nearest instance of feeding behavior, defined as biting or mastication by either one or both rats before the USV (Fig. 4*B*). In summary, 91.9% of 40 kHz flat USVs and 97.2% of 40 kHz inverted-U USVs were emitted within 0–1 s of a feeding behavior, whereas 49.0% of other USVs occurred within this time window. In contrast, 46.5% of other USVs were emitted >5 s after a feeding event. Pair-wise latency distributions of USVs relative to feeding onset further confirmed that the temporal linkage between feeding behavior and 40 kHz inverted-U and flat USVs was consistent across rat pairs (Extended Data Fig. 4-1). These results suggest that 40 kHz flat and inverted-U USVs are tightly coupled with chocolate-feeding behavior.

### Applicability of the logistic regression model and reproducibility of the 40 kHz flat and inverted-U USVs during chocolate consumption

We next validated the logistic regression model to distinguish between the “pre”- and “post”-type USVs in different rat pairs. We also investigated the association between the “post”-type, 40 kHz flat, and 40 kHz inverted-U USVs and chocolate consumption. We created 11 new rat pairs and included another group in which empty containers (without chocolate) were presented in the postperiod (empty group, 11 pairs; Fig. 5*A*). Furthermore, we included chocolate priming before the habituation sessions and increased the amount of chocolate used for priming (10 g × 2 d) for both chocolate and empty groups. The chocolate consumption of the chocolate group significantly increased across the three sessions (repeated-measures one–way ANOVA, *F*_(1.85,18.5)_ = 51.29; *p* < 0.0001; Fig. 5*B*). Both the empty and chocolate groups exhibited a larger number of 50 kHz USVs during the two habituation sessions and the three chocolate presentation sessions (Fig. 5*C*,*D*) than the previous dataset (Fig. 1*C*,*D*). We speculate that the increased number of USV calls reflects a heightened expectation level for the chocolate of rats, resulting from the additional amount provided during priming.

Next, we categorized all USVs detected in this test dataset as “pre”- and “post”-types using the classification model (Fig. 6*A*). “Pre”-type USVs were emitted in both the pre- and postperiods across all sessions in both empty and chocolate groups. In contrast, “post”-type USVs were rare during the preperiod in all sessions of both empty and chocolate groups. However, during the postperiod of the chocolate group, “post”-type calls were scarce in the habituation sessions but increased to significant levels during the chocolate sessions. Notably, even during the postperiod of empty group sessions in which chocolate was unavailable, USVs were rarely classified as “post”-type, indicating that “post”-type classifications were not simply driven by elapsed time within the session.

We then analyzed the spectrogram of USVs identified as “pre”-type and “post”-type in this new dataset. From each pair of rats in the chocolate group, we selected the top five USVs with the highest classification confidence (Fig. 6*B*; Extended Data Fig. 6-1) and evaluated them visually based on the conventional USV-type classification (Wright et al., 2010). The “pre”-type USVs with high confidence scores were mostly short or unclassifiable types, whereas most of the “post”-type USVs with high confidence scores were either flat or inverted-U types, representing 49 and 28%, respectively (Fig. 6*C*). We also confirmed that, in the test dataset, the principal frequency of “post”-type USVs was ∼40 kHz, which was significantly lower than that of “pre”-type USVs (Fig. 6*D*).

Next, we examined the proportion of 40 kHz flat and 40 kHz inverted-U type USVs among those classified as “pre”- and “post”-type by the model in the new dataset (Fig. 6*E*). The “pre”-type USVs contained few 40 kHz flat or 40 kHz inverted-U types. In contrast, among the USVs classified as “post”-type with high confidence (logistic regression score, >0.75), 52% were 40 kHz flat USVs, and 31% were 40 kHz inverted-U USVs. For USVs classified as “post”-type with low confidence (logistic regression score, between 0.5 and 0.75), 34% were 40 kHz flat USVs, and 9% were 40 kHz inverted-U USVs.

We then visually inspected all USVs in the new dataset to determine whether they belonged to the 40 kHz flat or 40 kHz inverted-U type. The results revealed that both 40 kHz flat and 40 kHz inverted-U USVs rarely occurred when chocolate was unavailable, whereas they were emitted specifically during the postperiod of the chocolate presentation sessions in the chocolate group (Fig. 7*A*). We then examined whether the 40 kHz flat and inverted-U USVs were associated with chocolate consumption in the new dataset (Fig. 7*B*), as depicted in Figure 4*B*. Again, 93.7% of 40 kHz flat USVs and 96.6% of 40 kHz inverted-U USVs were emitted within 0–1 s of a feeding behavior, whereas 39.6% of other USVs occurred within this time window. In contrast, 59.3% of other USVs were emitted >5 s after a feeding event. In this test dataset, pair-wise latency distributions of USVs relative to feeding onset again showed a consistent temporal linkage between feeding behavior and 40 kHz inverted-U and flat USVs, which was consistent across rat pairs (Extended Data Fig. 7-1). These results further confirmed the close association between 40 kHz flat and inverted-U USVs and chocolate-feeding behavior.

### Effect of opioid blocker naloxone on 40 kHz flat and inverted-U USVs during chocolate consumption

We next examined the influence of opioids on “post”-type, 40 kHz flat, and 40 kHz inverted-U USVs, given that opioid receptor blockers reduce “liking” reactions in the taste reactivity test (Parker et al., 1992). Following three chocolate presentation sessions with the chocolate group, the naloxone injection experiment was conducted with the same 11 rat pairs (Fig. 8*A*). The rats were administered either naloxone (5 mg/kg) or vehicle saline subcutaneously, and after 10 min, USV recordings and chocolate presentation were carried out in the same manner as in the previous sessions. Naloxone did not significantly affect the latency to initiate chocolate consumption (Wilcoxon test, *p* = 0.33; Fig. 8*B*) but significantly decreased chocolate consumption (paired *t* test, *t*_(10)_ = 6.93; *p* < 0.0001; Fig. 8*C*).

Overall, USV emissions were reduced by naloxone during both the pre- and postperiods (Wilcoxon test, *p* = 0.004 for preperiod, *p* = 0.02 for postperiod; Fig. 8*D*). When examining the effect of naloxone on “pre”- and “post”-type USV emissions, both types showed a significant decrease in the preperiod (*p* = 0.0010 and 0.008, respectively; Extended Data Fig. 8-1*A*). In the postperiod, both “pre- and “post”-types also were significantly reduced by naloxone (*p* = 0.02 and 0.015, respectively; Fig. 8*E*). Furthermore, we analyzed the effect of naloxone on 40 kHz flat and inverted-U USV emissions. In the preperiod, naloxone did not lead to a significant decrease in 40 kHz flat and inverted-U USVs (*p* = 0.50 for flat USVs, not applicable for inverted-U USVs) but decreased other types of USVs (*p* = 0.003; Extended Data Fig. 8-1*B*). In contrast, in the postperiod, naloxone significantly reduced 40 kHz flat, 40 kHz inverted-U, and other types of USVs (*p* = 0.02 for flat USVs. *p* = 0.008 for inverted-U USVs; *p* = 0.012 for other types of USVs; Fig. 8*H*).

Because naloxone significantly reduced chocolate intake (Fig. 8*C*), we normalized postperiod USV counts to the amount of chocolate consumed (calls per gram of intake) to control for this confounding factor. Using this intake-normalized metric, we found that model-classified “post”-type and 40 kHz inverted-U calls were significantly reduced by naloxone (Wilcoxon test, *p* = 0.03 and 0.008, respectively; Fig. 8*G*,*J*), whereas “pre”-type and 40 kHz flat calls were no longer significantly reduced (*p* = 0.31 and 0.12, respectively; Fig. 8*F*,*I*), and other call subtypes were unaffected (*p* = 0.10; Fig. 8*H*). These results indicate that while naloxone reduced overall USV emission, only the suppression of 40 kHz inverted-U calls persisted after normalizing by intake.

### Higher occurrence of 40 kHz inverted-U during palatable chocolate feeding compared with regular food

The results described above suggest that “post”-type USVs, including 40 kHz flat and 40 kHz inverted-U subtypes, are associated with chocolate consumption. However, it remains unclear whether these USVs are specifically linked to palatable food consumption or instead reflect more general affective or behavioral states associated with feeding. To address this point, we examined USVs emitted during regular chow feeding and compared them with those emitted during chocolate feeding (Fig. 9).

USV recordings were conducted at ZT12–14 (immediately after light off), when rats reliably consume regular chow and chocolate without fasting (Fig. 9*A*; Zorrilla et al., 2005). A total of eight pairs of rats (16 rats) were pair-housed and first underwent a priming session with chocolate, followed by two habituation sessions in a recording chamber. Subsequently, rats were tested in sessions in which either regular chow or chocolate was presented, with the order counterbalanced across animals. Each session lasted 2 h to ensure sufficient feeding opportunities, and no food deprivation was applied. Under these conditions, total food intake was comparable between the two foods (paired *t* test, *p* = 0.81; Fig. 9*B*).

Despite similar intake, 40 kHz inverted-U USVs were significantly more frequent during chocolate consumption than during regular chow consumption (paired *t* test, *p* = 0.009; Fig. 9*D*), whereas 40 kHz flat USVs and other subtypes were observed at comparable levels for both foods (*p* = 0.37 for 40 kHz flat USVs and 0.31 for other types; Fig. 9*C*,*E*). Palatability of chocolate was subsequently confirmed using a two-food choice test, in which 15 of 16 rats showed a clear preference for chocolate over regular chow (Fig. 9*F*). These results demonstrate that inverted-U calls are not simply a general feature of consummatory behavior but are selectively enhanced during consumption of a more palatable food.

## Discussion

In this study, we investigated whether specific subtypes of USVs are emitted by rats when they consume palatable food (chocolate) and whether these USVs can be objectively classified using machine learning. Through analysis of acoustic parameters of USVs and the application of a logistic regression model, we found that rats emit distinct USV subtypes when given access to chocolate, particularly 40 kHz inverted-U subtypes, in addition to 40 kHz flat subtypes that are also observed during consumption of standard food pellets. Direct comparison between regular chow and chocolate under intake-matched conditions demonstrated that 40 kHz inverted-U USVs are selectively associated with palatable feeding rather than with consummatory behavior per se. These findings raise the possibility that rat USVs are modulated by food palatability. Moreover, the emission of 40 kHz inverted-U USVs was preferentially reduced following administration of the opioid receptor antagonist naloxone, suggesting that the opioid-regulated reward system contributes to their emission.

### Modulation of USVs by food palatability

Our findings regarding the emission of 40 kHz ranged USVs during chocolate consumption are partially consistent with previous reports (Takahashi et al., 2010; Champeil-Potokar et al., 2023). According to Takahashi et al., USVs in Sprague Dawley rats can be classified into three main clusters based on frequency range and call duration (Takahashi et al., 2010). USVs emitted during the consumption of standard laboratory chow generally fall within the 40 kHz range (30–50 kHz). The spectrogram shape of these USVs is predominantly flat, with the inverted-U type being rarely observed (accounting for <1% relative to flat USVs). In our study, the occurrence of inverted-U USVs during chocolate consumption was markedly higher, representing ∼48% (training dataset) and 45% (test dataset) of the number of flat USVs. Champeil-Potokar et al. reported that Wistar rats emit USVs while masticating food (Champeil-Potokar et al., 2023). In their study, using standard laboratory chow, the predominant USV type was flat in the 40 kHz range (35–50 kHz), with very few inverted-U types observed. In our spectrogram analysis, we observed similar overlap between sounds produced during chocolate mastication and USVs, identifying both flat and inverted-U USVs within the 40 kHz range (30–50 kHz). These results suggest that rats typically produce flat USVs in the 40 kHz range while feeding and that palatability of the food may modulate their vocalizations from flat to inverted-U shape in the spectrogram.

Importantly, our analyses also revealed substantial variability in 50 kHz call rates and relative prevalences of 50 kHz call subtypes across rat pairs, consistent with prior studies (Sundarakrishnan and Clarke, 2022). Nevertheless, under chocolate-available conditions, all rat pairs emitted at least one “post”-type USV across sessions. This indicates that “post”-type calls were detected even in rat pairs with relatively low overall call rates and that the predictive parameters identified by the model are not limited to high-calling pairs. In addition, with respect to the 40 kHz inverted-U subtype, 17 of the total 21 rat pairs indeed emitted this call under chocolate-available conditions. Thus, although individual differences in 50 kHz USV emission are clearly present, the key call subtypes highlighted in this study are not restricted to a few exceptional high-calling pairs.

One alternative interpretation is that USVs, particularly 40 kHz inverted-U types, might reflect social dominance or submission rather than palatable feeding as the present paradigm possibly constitutes a form of food competition. While social inequality has been shown to modulate USVs in other paradigms (Okabe et al., 2023), the present data are more consistent with an association between 40 kHz inverted-U USVs and palatable feeding itself. In all rat pairs, both animals consumed chocolate during the recording period and sustained monopolization of food by a single animal was not observed. Moreover, inverted-U calls occurred, while both rats were actively feeding, arguing against an interpretation based on frustration or exclusion from food access.

Evidence from food-associated calls in rhesus macaques also supports the idea that food palatability influences vocal expression (Hauser and Marler, 1993a,b). Rhesus macaques exhibit different types of vocalizations when encountering rare, highly preferred foods, such as coconut, compared with standard chow. These cross-species observations raise the possibility that vocalizations may represent affective aspects of feeding, including palatability. At the same time, differences in USV profiles between pre- and postperiods may also reflect other feeding-related affective processes, such as anticipation or frustration during waiting, and relief from frustration rather than positive affect associated with consumption per se. Disentangling these components remains a general challenge in affective neuroscience and cannot be fully resolved within the present experimental framework. Future studies incorporating explicit omission or delay paradigms, as well as systematic manipulation of food palatability (e.g., tasteless, low-nutrient, high-fat, or high-sugar), will be required to more precisely dissociate these affective processes.

### Machine learning–based classification of USV subtypes

In the present study, we trained a classification model based on pre- versus postchocolate presentation periods, allowing us to identify USVs associated with chocolate availability. This approach revealed a subtype of USVs categorized as “post”-type, which included both 40 kHz flat and 40 kHz inverted-U calls. Importantly, while the classifier alone does not fully distinguish palatability from general feeding-related states, additional manual classification (40 kHz flat vs inverted-U) by human raters enabled further dissociation within the “post”-type calls defined by the classifier. Specifically, 40 kHz inverted-U calls were selectively enhanced during chocolate consumption, whereas 40 kHz flat calls were equally observed during both chocolate and regular chow feeding. Together, these findings suggest that machine learning–assisted classification, combined with human-guided subtype analysis, can reveal distinct components of feeding-related USVs, including those associated with palatability.

A methodological advancement in our study is the use of machine learning to classify USV subtypes emitted before and after chocolate presentation periods based on their spectrogram characteristics. We utilized a logistic regression model for supervised binary classification, which facilitated confidence scoring that helped identify USV subtypes related to chocolate consumption. Conventional methods for USV classification largely rely on manual visual inspection. In contrast, our logistic regression model, trained on 11 spectrogram parameters, achieved high-throughput classification of USVs with an AUC of 0.78. This approach facilitated a more objective and consistent analysis across different rat pairs and experimental sessions, reducing human error and interobserver variability.

To evaluate the effectiveness of our model, we created another logistic regression model using a randomized training dataset with shuffled pre- and postlabeling and compared the AUC values. This approach yielded a model with an AUC close to the chance level (0.51), indicating the shuffled labels eliminated the discriminative structure present in the original data. Although the model with the randomized dataset achieved a seemingly higher accuracy (83.1%) than our original model (82.5%), this higher accuracy was expected given the imbalance in the ratio of pre- and postlabeled USVs in the training dataset (prelabel, 5,893 calls; postlabel, 2,016 calls). In fact, the model with the randomized dataset classified all USVs in the training dataset as “pre”-type. These results suggest that USVs in the postperiod have distinct spectrograms compared with those in the preperiod and that our model successfully utilized these differences to classify USVs into the “pre”- and “post”-types. Although the number of calls used for training was sufficient to detect robust differences and was validated using an independent test dataset, larger datasets will be valuable in future studies to further refine machine learning–based classification of USVs.

A limitation of the current model training is that the training dataset did not include a no-chocolate control condition. This limitation was partly addressed using the independent test dataset, which demonstrated that “post”-type classifications were contingent on chocolate availability rather than elapsed time within the session. Notably, when the trained classifier was applied to the independent test dataset, USVs emitted during the postperiod of no-chocolate sessions were rarely classified as “post”-type, indicating that the classifier did not simply capture time-dependent changes within a session. Because the classifier was trained using pre- and postperiod labels, the terms “pre” and “post” may be misleading if interpreted strictly as temporal descriptors. Instead, these findings suggest that the classification more accurately reflects chocolate availability rather than temporal position within a session.

Our model identified call length and tonality as the most important features distinguishing USVs emitted before and after chocolate presentation. During the postperiod, USVs were generally longer than those in the preperiod. Furthermore, rats remained still while feeding chocolate, which reduced movement-associated broadband noise ∼5 kHz and resulted in higher tonality, specifically in the signal-to-noise ratio. Although tonality contributed to the classifier, it was not interpreted as a direct marker of affective state but rather as reflecting behavioral context. Importantly, our main conclusions are supported by not only the machine learning–based classification but also spectrographic USV subtyping and frequency. Thus, our model effectively captured these USV characteristics and related behavioral states, ensuring the reliability of machine learning for USV classification.

### Roles of opioids in USV emissions during palatable feeding and their implications for positive emotional states

In this study, we found that systemic administration of naloxone significantly reduced the emission of both 40 kHz flat and 40 kHz inverted-U USVs during chocolate consumption while having less impact on other USV types. This suggests that the emission of these two subtypes is modulated by the endogenous opioid system. The opioid system is well known to contribute to positive emotional states in animals (Emery and Akil, 2020; Murata et al., 2024). In particular, during the hedonic “liking” reactions observed in the taste reactivity test, opioid receptor antagonists reduce characteristic responses such as rhythmic tongue protrusions (Parker et al., 1992).

We used a relatively high dose of naloxone (5 mg/kg) to ensure robust suppression of opioid-dependent hedonic processes. Naloxone did not significantly alter the latency to initiate chocolate consumption, indicating that general mobility, sensory recognition of chocolate, and the initial motivation to approach and ingest the food were largely preserved. Nevertheless, we acknowledge that the use of a relatively high naloxone dose may raise concerns regarding dose-dependent specificity. The effects of high naloxone dose cannot be assumed to be restricted to classical opioid receptors. Specifically, the engagement of additional nonopioid mechanisms (e.g., Toll-like receptor 4; Hutchinson et al., 2012) cannot be fully excluded. Further studies employing lower doses and dose–response designs will be required to assess the specificity of opioid involvement in feeding-related USVs.

Previous studies have also shown that administration of psychostimulants and opioid blockers modulates the emission of 50 kHz USVs and their subtypes, implicating contributions of opioids to vocalization patterns linked to positive motivational states (Burgdorf et al., 2001; Wöhr and Schwarting, 2009; Buck et al., 2014; Brudzynski, 2015). Our findings extend this framework by demonstrating that palatable food–associated 40 kHz inverted-U USVs are likewise sensitive to opioid receptor blockade, indicating that these vocalizations may serve as behavioral markers of positive emotional states during feeding. Future research should aim to identify the specific neural circuits responsible for the generation of these opioid-sensitive USVs.

### Limitations and future directions

Our study was designed to minimize the influence of social and sexual factors, focusing specifically on a simplified and controlled feeding context. To this end, we used male rat dyads that had been pair-housed for weeks, allowing the establishment of a stable social relationship prior to experimentation. While this design allowed us to isolate chocolate-feeding–related USV patterns under controlled conditions, it also raises the question of how generalizable these findings are to more naturalistic or socially complex environments. Future studies should explore whether similar USV patterns, particularly the 40 kHz inverted-U subtype, are observed under more ecologically valid conditions, including group housing, individually housed (solo) rats, or female subjects.

One technical limitation concerns the possibility of false negatives at the detection of USV events by DeepSqueak. Because DeepSqueak was used as the initial detector, some USV calls may have been missed during automated detection. To address this concern for trill calls in particular (Wright et al., 2010), which are reported to be challenging for automated contour-based classification, we performed visual verification across the chocolate consumption sessions of all 10 rat pairs in the training dataset and found that DeepSqueak detected 99.1% of manually identified trill calls (see Materials and Methods). Importantly, our analyses focused on the classification of detected calls rather than absolute call counts. Therefore, any remaining missed events would be unlikely to affect our main conclusions regarding the selective emergence of specific call types, including the “post”-type and the 40 kHz inverted-U type, during chocolate feeding.

A related methodological consideration concerns the broadband noise observed ∼5 kHz in our recordings, the magnitude of which appeared to correlate with the level of animal activity. While this temporal association suggests a mechanical source related to animal activity, such as friction with the bedding, contact between the animal and recording apparatus, or vibration of cage components, an electromagnetic origin (e.g., aliasing from radio-frequency transmitters or radiation from nearby electrical devices) cannot be entirely excluded. The precise origin of this noise has not been definitively identified and would require more detailed investigation.

Another important limitation concerns the identification of the vocalizing individual. Although our behavioral analysis demonstrated that the inverted-U and flat USVs were more tightly linked to chocolate-feeding behavior than other USVs, we could not distinguish which of the two rats in a pair produced each vocalization. As such, the current data cannot rigorously determine whether the emitting rat was directly engaged in feeding at the moment of USV emission. To establish whether the 40 kHz inverted-U USV serves as a real-time emotional marker of feeding pleasure, future work must incorporate improved tracking of individual vocalizers and concurrent behavioral states.

The identification of a novel 40 kHz inverted-U USV subtype associated with chocolate consumption adds to our understanding of the biological significance of USV diversity. As shown in our data, these USVs were tightly linked to chocolate-feeding behavior and were suppressed by opioid receptor blockade, suggesting their potential involvement in affective processes related to palatable food intake. USVs are widely recognized as signals for social communication, and in this context, it is worth considering whether the 40 kHz inverted-U subtype identified here may also play a communicative role (Wöhr, 2017). Hauser and Marler reported that rhesus monkeys produced distinct vocalizations when discovering highly palatable foods, which functioned as honest signals that reduced aggression from conspecifics and increased food access (Hauser and Marler, 1993a,b). In rodents, social transmission of food preferences has been demonstrated: observer rats acquire a preference for foods consumed by demonstrators, primarily through olfactory cues (Galef and Whiskin, 1992; Bessières et al., 2017). These findings raise the intriguing possibility that food-related USVs—such as the 40 kHz inverted-U subtype—may not only reflect internal affective states but also broadcast information about the presence or value of palatable food to nearby conspecifics. Future studies are needed to test this hypothesis by examining the emission and reception of such USVs under socially dynamic conditions.

In summary, our findings suggest that specific USV subtypes, particularly the 40 kHz inverted-U type, may serve as acoustic signatures during palatable food consumption. These vocalizations are modulated by the opioid system and can be efficiently detected using machine learning. Extending this framework to other sensory modalities or types of reward will be essential for evaluating the generalizability and neurobiological basis of vocal-affective expressions in animals.

## Synthesis

Reviewing Editor: Maithe Arruda-Carvalho, University of Toronto Scarborough Decisions are customarily a result of the Reviewing Editor and the peer reviewers coming together and discussing their recommendations until a consensus is reached. When revisions are invited, a fact-based synthesis statement explaining their decision and outlining what is needed to prepare a revision will be listed below. The following reviewer(s) agreed to reveal their identity: NONE. Note: If this manuscript was transferred from JNeurosci and a decision was made to accept the manuscript without peer review, a synthesis may not be available.

The reviewers recognized and appreciated the considerable revisions made to the manuscript, as well as the authors' systematic and careful responses to reviewer comments. Nevertheless, there remains one major concern to be addressed by the authors, in addition to some minor suggestions listed below.

The main concern centers on determining whether their analysis might be missing an important category of 50-kHz vocalizations, the "trill" call as defined by Wright et al 2010. DeepSqueak is known to fail to recognize this type of vocalization, and trill calls were mostly absent in their results (Fig. 3C) - very surprising given that the literature typically reports trill calls among the most prevalent across a wide variety of rewarding contexts. The authors have added a new paragraph (lines 775-781) pointing out that DeepSqueak can fail to identify some 50-kHz calls, but critically, they do not mention trill calls. The potential omission of trill calls is very important because multiple reports suggest that trill calls, more than any other call subtype, reflect positive affect. Therefore, when looking for call subtypes that might change during chocolate consumption (a main aim of this study), the trill call subtype would likely be the first to come to mind.

To address this, the authors must visually identify occurrences of the trill call subtype in their original sound recordings, and quantify the percentage of which was missed by DeepSqueak. If they find that DeepSqueak has missed only a small proportion (<20%) of trill calls, then it would be sufficient to report, in Methods or Results, that human raters detected XXX trill calls, of which DeepSqueak recognized Y%. This should also be mentioned as a limitation in the Discussion.

If, on the other hand, DeepSqueak missed more than ~20% of trill calls, the authors could (1) add the missing trill calls to the data set and then rerun all the relevant analysis, or (2) run an additional analysis of only trill calls, asking whether their relative prevalence increased upon chocolate consumption, compared to the preceding period where chocolate was unavailable, and also compared to the chow-consumption condition. Given that this is a methods manuscript, the second approach would be acceptable.

Additional minor suggestions/concerns:

- Extended Figure 8-1 panel A. Is the comparison between saline and naloxone really significant in the 'Post'-type condition? Is it possible that the analysis was not done with rat pair being the experimental unit, i.e. a case of pseudoreplication as in the first manuscript submission?

- Line 270. Source of 5-kHz noise. The authors suggest that this is likely caused by the rat disturbing the bedding while moving around the test box. Extended Fig. 3-1 shows many spectrograms extracts. The noise is concentrated in at 5 kHz both in the pre-chocolate and "post" period. This "tonal" noise is unlikely to come from bedding, especially since it often occurred in the absence of broad-spectrum noise. Instead, it might be from aliasing from a radiofrequency transmitter, or radiation from some electrical device in the vicinity. Therefore, the suggestion that bedding could explain the focused 5-kHz noise should be removed.

- Extended Figs. 3-1 and 6-1. The 100-ms time bars may be shorter than they should be, given that many categories of 50-kHz calls tend to be less than 50 ms long.

- Please explain, in each figure legend, the meaning of Hab1, Hab2, Choc1, etc.

- Incorporation of repeated measures aspect of the design into statistical analyses. This has been done only in part. For example, Fig. 5C and 5D should use Friedman test rather than Kruskal-Wallis. Same comment about Figures 6 and 7. This will also change the statistical results reported in the Extended Data documents. As an example, Extended Data Figure 2-2 suggests Mann-Whitney tests were used in Fig. 2. Why not a test appropriate for repeated measures design?

- Please explain in the Statistical analysis section what kind of repeated measures ANOVA was used, to clarify why the degrees of freedom values are not integers.

- How was the y-axis variable in Fig. 6C computed, and why is there no error bar? Please make the y-axis label more explicit, rather than just "%". What does this percentage represent? For example, are you referring to the prevalence of each call subtype, as a percentage of the total number of 50-kHz calls?

- Line 403. Given the experimental design, rat pair is the correct experimental unit, and anything else would be pseudoreplication (as the authors acknowledge). Therefore, it is incorrect to say that making rat pair the experimental unit was done "to provide a more conservative statistical assessment". The words "more conservative" should be changed to "adequately conservative".

Minor text changes:

- Change all occurrences of "not available" to "unavailable".

- Line 156: "The training dataset was designed to prioritize sessions with chocolate availability..." Rephrase for clarity. It is unclear what "prioritize" means. Do you mean that the training dataset comprised only sessions when chocolate was given?

- Line 178: "to eliminate variability" would be better phrased as "to avoid variability"

- Line 250: This would read better as "starting USV recording by providing rats"

- Line 258: "hosed" should read "housed"

- Line 469 should read as follows: "...40-kHz inverted-U USVs were rarely..."

- Line 539 should read "...USVs, which was consistent across rat pairs..."

- Line 957-958: There is a minor error

- Lines 327, 462, 523, 1008, 1061, and possibly elsewhere. The "<" symbol is in the wrong place. For example, "5<" should read "<5".

- Lines 1072, 1075 and possibly elsewhere: The semicolon should be a colon.

- Please consider, if the author instructions allow, merging the extended data into a smaller number of files.

- The Extended Data file names all contain "video", yet none are video files. Perhaps this was added by the submission system, but it seems strange.

- Extended Data Figure 4-2. Please add the statistical results for "other type USVs"?

- Extended Figure 8-1 panel B: What does "NA" mean?
